# Fetal and maternal NLRP3 signaling is required for preterm labor and birth

**DOI:** 10.1172/jci.insight.158238

**Published:** 2022-08-22

**Authors:** Kenichiro Motomura, Roberto Romero, Jose Galaz, Li Tao, Valeria Garcia-Flores, Yi Xu, Bogdan Done, Marcia Arenas-Hernandez, Derek Miller, Pedro Gutierrez-Contreras, Marcelo Farias-Jofre, Siddhesh Aras, Lawrence I. Grossman, Adi L. Tarca, Nardhy Gomez-Lopez

**Affiliations:** 1 Perinatology Research Branch, Division of Obstetrics and Maternal-Fetal Medicine, Division of Intramural Research, Eunice Kennedy Shriver National Institute of Child Health and Human Development, National Institutes of Health, US Department of Health and Human Services (NICHD/NIH/DHHS), Detroit, Michigan, USA, and Bethesda, Maryland, USA.; 2 Department of Obstetrics and Gynecology, Wayne State University School of Medicine, Detroit, Michigan, USA.; 3 Department of Obstetrics and Gynecology, University of Michigan, Ann Arbor, Michigan, USA.; 4 Department of Epidemiology and Biostatistics, Michigan State University, East Lansing, Michigan, USA.; 5 Center for Molecular Medicine and Genetics, Wayne State University, Detroit, Michigan, USA.; 6 Detroit Medical Center, Detroit, Michigan, USA.; 7 Division of Obstetrics and Gynecology, Faculty of Medicine, Pontificia Universidad Católica de Chile, Santiago, Chile.; 8 Department of Computer Science, Wayne State University College of Engineering, Detroit, Michigan, USA.; 9 Department of Biochemistry, Microbiology, and Immunology, Wayne State University School of Medicine, Detroit, Michigan, USA.

**Keywords:** Immunology, Reproductive Biology, Cellular immune response, Molecular biology, Obstetrics/gynecology

## Abstract

Preterm birth is the leading cause of neonatal morbidity and mortality worldwide. One of every 4 preterm neonates is born to a mother with intra-amniotic inflammation driven by invading bacteria. However, the molecular mechanisms underlying this hostile immune response remain unclear. Here, we used a translationally relevant model of preterm birth in *Nlrp3*-deficient and -sufficient pregnant mice to identify what we believe is a previously unknown dual role for the NLRP3 pathway in the fetal and maternal signaling required for the premature onset of the labor cascade leading to fetal injury and neonatal death. Specifically, the NLRP3 sensor molecule and/or inflammasome is essential for triggering intra-amniotic and decidual inflammation, fetal membrane activation, uterine contractility, and cervical dilation. NLRP3 also regulates the functional status of neutrophils and macrophages in the uterus and decidua, without altering their influx, as well as maternal systemic inflammation. Finally, both embryo transfer experimentation and heterozygous mating systems provided mechanistic evidence showing that NLRP3 signaling in both the fetus and the mother is required for the premature activation of the labor cascade. These data provide insights into the mechanisms of fetal-maternal dialog in the syndrome of preterm labor and indicate that targeting the NLRP3 pathway could prevent adverse perinatal outcomes.

## Graphical abstract

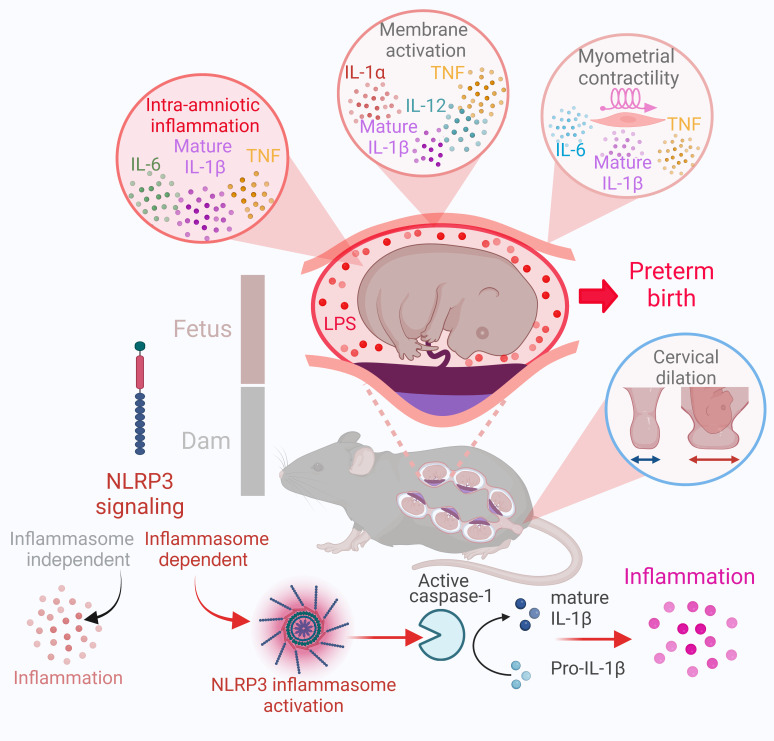

## Introduction

Preterm birth, delivery prior to 37 weeks of gestation, is the leading cause of neonatal morbidity and mortality as well as the second greatest cause of childhood death below the age of 5 (1). Approximately 15 million preterm neonates are born every year, with North America having one of the highest rates (2). Two-thirds of all preterm births occur after the spontaneous onset of preterm labor, a syndrome of multiple pathological processes, of which microbe-associated inflammation in the amniotic cavity (i.e., intra-amniotic infection) is a well-established cause (3). Indeed, 1 of every 4 preterm infants is born to a mother with intra-amniotic infection (4). This condition is largely subclinical and frequently results from the invasion of the amniotic cavity by microorganisms ascending from the lower genital tract (5, 6). Invading microorganisms are predominantly bacteria (e.g., genital mycoplasmas and Gram-positive and -negative bacteria) that possess pathogen-associated molecular patterns, which are sensed by host pattern recognition receptors (7), inducing activation of the innate immune system and a subsequent inflammatory milieu in the amniotic cavity (8, 9). This immune response comprises proinflammatory cytokines released by the surrounding fetal tissues as well as infiltrating immune cells (10–15). Therefore, elucidating the mechanisms underlying such an inflammatory response is critical for the development of novel therapeutic strategies to prevent preterm birth and subsequent adverse neonatal outcomes.

The NLRP3 inflammasome is a multiprotein complex that mediates the activation of caspase-1 and subsequent processing and release of IL-1β (16, 17), a cytokine that has been described as an essential facilitator of human parturition (18, 19). IL-1β is synthesized as a zymogen and therefore requires cleavage to exhibit its bioactivity (20). Clinical studies have suggested that the NLRP3 inflammasome is implicated in the processing of mature IL-1β in the chorioamniotic membranes (fetal tissues surrounding the conceptus) of women who underwent spontaneous preterm labor and birth (21–23). Moreover, using animal models of ultrasound-guided intra-amniotic inflammation, we showed that a microbial product (LPS) activates the NLRP3 inflammasome in the fetal membranes (murine tissues resembling human chorioamniotic membranes) (24). Yet, the complex pathway of parturition comprises multiple well-orchestrated biological processes, including fetal membrane activation, uterine contraction, and cervical dilation, as well as the triggering of an immune response in the decidual tissues (3, 25, 26). Therefore, the mechanisms of parturition require cooperation between the mother, who possesses the effector organs (myometrium, cervix, and decidua), and the fetus (fetal membranes). However, whether the NLRP3 inflammasome is implicated in such fetal and maternal compartments is largely unknown.

In the current investigation, we used multiple mechanistic approaches to fully appreciate the fetal and maternal contribution to NLRP3 signaling in the pathophysiology of preterm birth induced by microorganisms invading the amniotic cavity. Specifically, fetal responses were evidenced as inflammation of fetal organs, the amniotic cavity, and the fetal membranes as well as alterations in hemodynamic parameters (i.e., heart rate and umbilical artery blood flow) in *Nlrp3*-deficient mice. Maternal responses included the activation of the common pathway of labor (indicated by uterine contractility and cervical dilation) and inflammatory processes in the decidua. Furthermore, immunophenotyping and RNA-Seq were performed to evaluate cellular innate immune responses (neutrophils and macrophages) in the uterus and decidua. Last, heterozygous mating and embryo transfer strategies were employed to unravel the individual fetal and maternal contributions to NLRP3 signaling leading to preterm birth and neonatal mortality.

## Results

### Nlrp3 deficiency protects against intra-amniotic inflammation-induced preterm birth and neonatal mortality.

**Figure 1:**
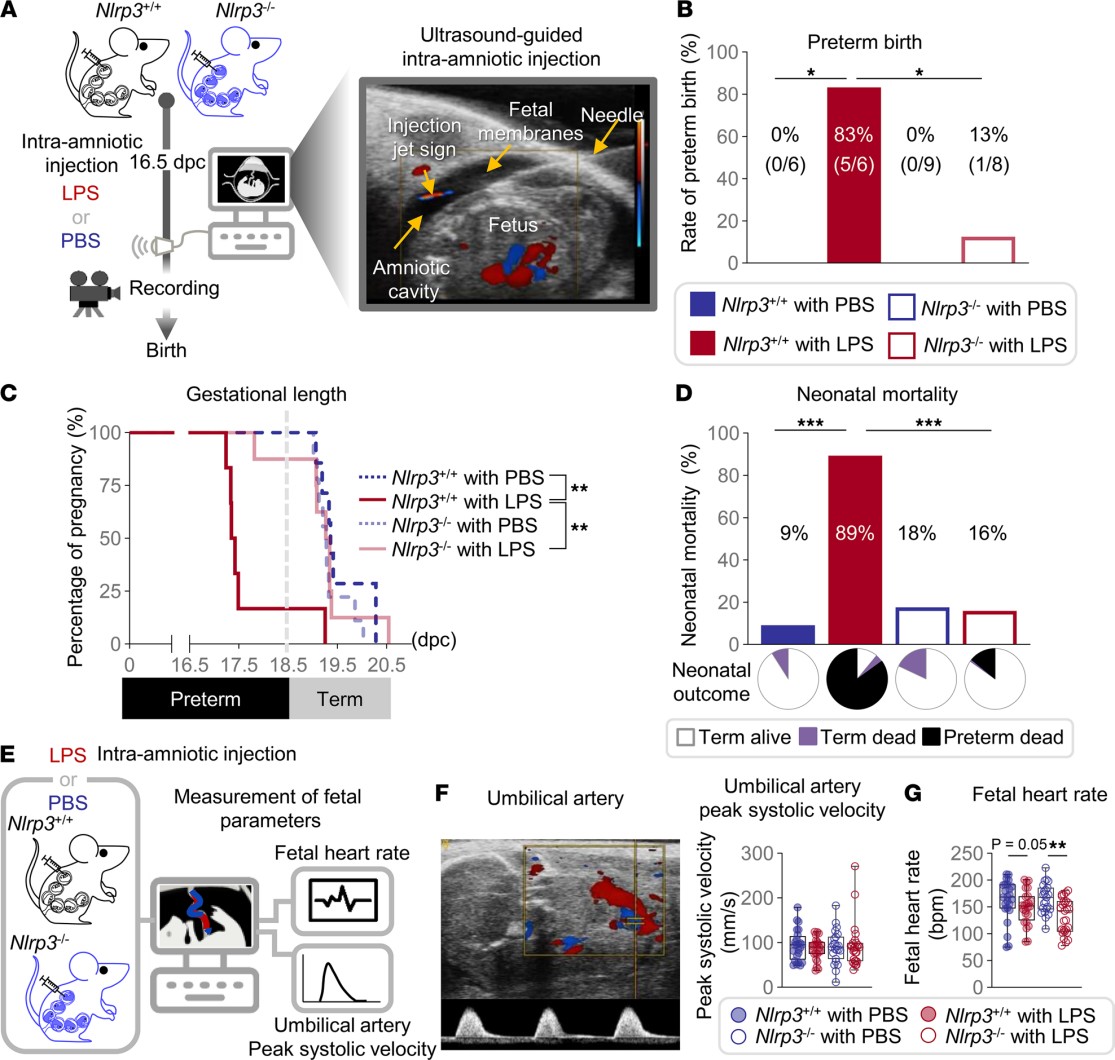
*Nlrp3* deficiency protects against intra-amniotic inflammation-induced preterm birth and neonatal mortality. (**A**) Pregnant *Nlrp3*^+/+^ and *Nlrp3*^–/–^ mice were intra-amniotically injected with LPS (100 ng/25 μL; *Nlrp3*^+/+^ mice, *n* = 6, *Nlrp3*^–/–^ mice, *n* = 8) or PBS (*Nlrp3*^+/+^ mice, *n* = 6, *Nlrp3*^–/–^ mice, *n* = 9) on 16.5 days post coitum (dpc). (**B**) Preterm birth rates. The *P* values were determined by Fisher’s exact test. (**C**) Gestational lengths. The *P* values were determined by Gehan-Breslow-Wilcoxon test. (**D**) Mortality rates of neonates. The pie charts represent neonatal outcomes as proportions (white: delivered at term and alive, purple: delivered at term and dead, black: delivered preterm and dead). The *P* values were determined by Fisher’s exact test. (**E**) Pregnant *Nlrp3*^+/+^ and *Nlrp3*^–/–^ mice were intra-amniotically injected with LPS (100 ng/25 μL; *Nlrp3*^+/+^ dams, *n* = 8, *Nlrp3*^–/–^ dams, *n* = 9) or PBS (*Nlrp3*^+/+^ dams, *n* = 8, *Nlrp3*^–/–^ dams, *n* = 8) in each amniotic sac under ultrasound guidance on 16.5 dpc. Doppler ultrasonography was performed on 17.5 dpc. (**F**) Representative Doppler ultrasound image of the umbilical artery and umbilical artery peak systolic velocity. (**G**) Fetal heart rate. Data are shown as box-and-whisker plots where midlines indicate medians, boxes indicate interquartile ranges, and whiskers indicate minimum and maximum values. The *P* values for the comparisons between PBS- and LPS-injected dams in each genotype were determined by Mann-Whitney *U* test. *P < 0.05; **P < 0.01; ***P < 0.001.

To investigate the contribution of the NLRP3 inflammasome to the onset of preterm labor leading to preterm birth, we used *Nlrp3*-sufficient (*Nlrp3*^+/+^) and -deficient (*Nlrp3*^–/–^) mice. A subclinical model of intra-amniotic inflammation induced by the ultrasound-guided intra-amniotic administration of LPS during late gestation (16.5 days post coitum, dpc) (27) was utilized (Figure 1A). As expected, the majority of *Nlrp3*^+/+^ mice injected with LPS delivered preterm, and those injected with PBS delivered at term (Figure 1B). Strikingly, most *Nlrp3*^–/–^ mice injected with LPS delivered at term, and only a fraction (13%, 1/8) delivered preterm (Figure 1B). Consistently, the gestational length of *Nlrp3*^–/–^ mice injected with LPS was significantly longer than that of LPS-injected *Nlrp3*^+/+^ mice (Figure 1C). These data show that *Nlrp3* deficiency protects against intra-amniotic inflammation-induced preterm birth.

Preterm neonates born to women with intra-amniotic infection require intensive care to mitigate the consequences of prematurity and the intra-amniotic inflammatory response (28), which can lead to devastating short- and long-term outcomes, including death, if not promptly managed (29). Therefore, we also evaluated neonatal mortality in *Nlrp3*^+/+^ and *Nlrp3*^–/–^ mice injected with LPS. Most neonates born to *Nlrp3*^+/+^ mice injected with LPS were delivered preterm and died shortly after birth, whereas those born to PBS-injected dams were viable and delivered at term (Figure 1D). By contrast, the majority of neonates born to *Nlrp3*^–/–^ mice injected with LPS or PBS were delivered at term (Figure 1D) and thrived into infancy. These results indicate that *Nlrp3* deficiency attenuates the deleterious neonatal effects induced by intra-amniotic inflammation.

### Nlrp3 deficiency disrupts LPS-induced inflammatory responses in the amniotic cavity and fetal tissues.

Intra-amniotic inflammation harms fetal well-being (30, 31). This concept is supported by mechanistic evidence demonstrating that antenatal exposure to inflammation causes fetal bradycardia and alters fetal-placental circulation (i.e., umbilical artery blood flow) (32, 33). Therefore, using high-resolution Doppler ultrasound to assess the umbilical artery, we evaluated whether the intra-amniotic administration of LPS alters fetal parameters and whether *Nlrp3* deficiency could protect the fetus against such alterations (Figure 1E). Fetuses of *Nlrp3*^+/+^ and *Nlrp3*^–/–^ mice did not display alterations in umbilical artery blood flow (Figure 1F). Yet, fetuses from *Nlrp3*^+/+^ mice injected with LPS were bradycardic compared with normocardic controls (Figure 1G). Such complication was more evident in fetuses of *Nlrp3*^–/–^ mice injected with LPS (Figure 1G), which led us to hypothesize that LPS triggers *Nlrp3*-dependent and -independent intra-amniotic and fetal inflammatory responses. In addition, ultrasound evaluation of the placenta revealed that there were no differences in placental thickness, diameter, or area between PBS- and LPS-injected *Nlrp3^–/–^* mice (Supplemental Figure 1, A–D; supplemental material available online with this article; https://doi.org/10.1172/jci.insight.158238DS1). Moreover, no changes in fetal and placental weights were found (Supplemental Figure 1, E–G).

**Figure 2:**
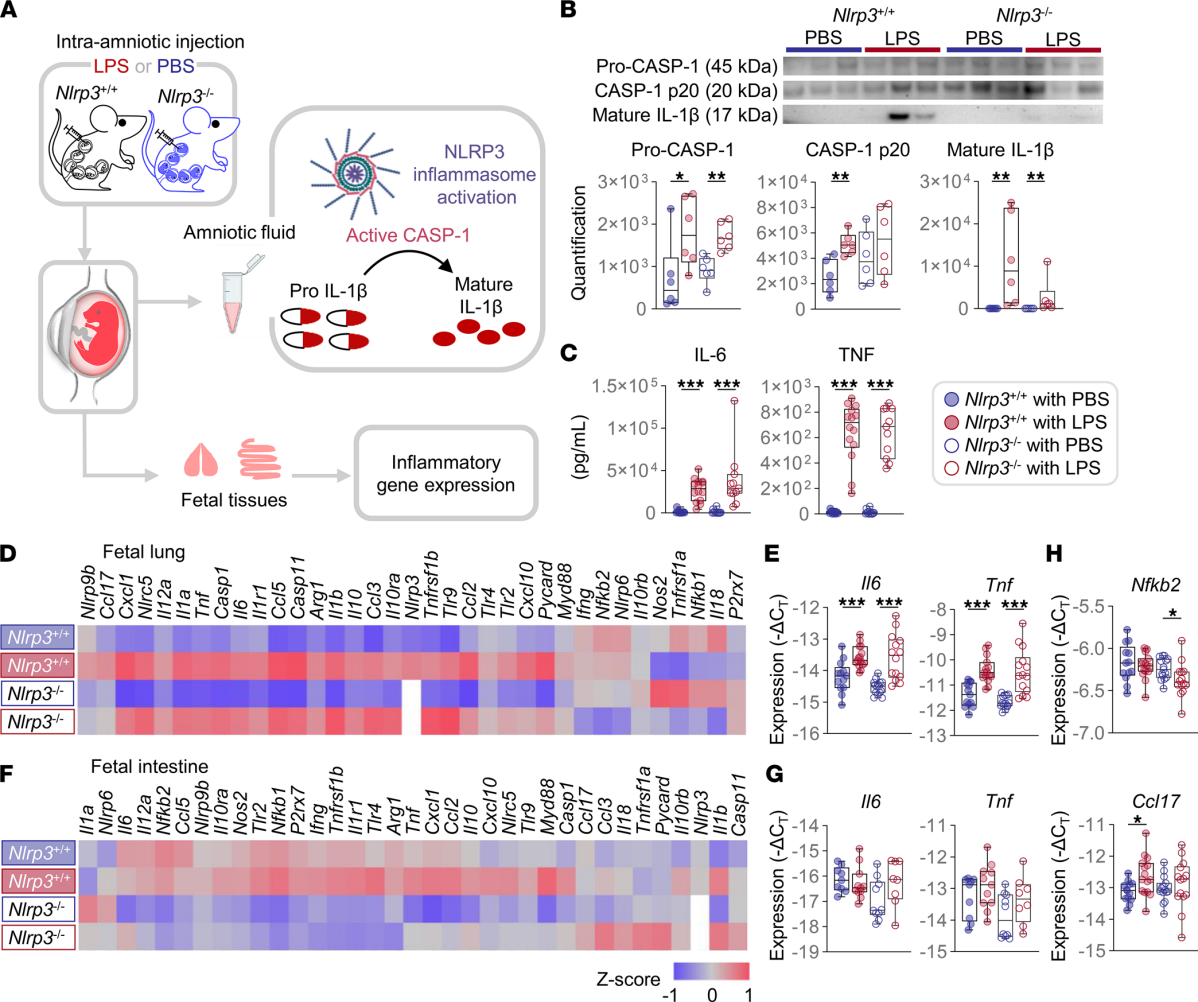
*Nlrp3* deficiency disrupts LPS-induced inflammatory responses in the amniotic cavity and fetal tissues. (**A**) Pregnant *Nlrp3*^+/+^ and *Nlrp3*^–/–^ mice were intra-amniotically injected with LPS (100 ng/25 μL) or PBS on 16.5 days post coitum (dpc). The collection of amniotic fluid, fetal lung, and fetal intestine was performed on 17.5 dpc. A representative diagram of the NLRP3 inflammasome activation pathway is shown. (**B**) Immunoblotting of pro-CASP, CASP-1 p20, and mature IL-1β in amniotic fluid (*n* = 6 per group). The immunoblotting was performed in 2 separate gels (*n* = 3 per group in each gel). Representative blot images were shown. (**C**) Amniotic fluid concentrations of IL-6 and TNF (*n* = 11–14 per group). (**D**) Heatmap visualization of inflammatory gene expression in the fetal lung (*n* = 13–15 per group). (**E**) Gene expression of *Il6* and *Tnf* in the fetal lung. (**F**) Heatmap visualization of inflammatory gene expression in the fetal intestine (*n* = 8–11 per group). (**G**) Gene expression of *Il6* and *Tnf* in the fetal intestine. (**H**) Gene expression of *Nfkb2* and *Ccl17* in the fetal lung. Data are shown as box-and-whisker plots where midlines indicate medians, boxes indicate interquartile ranges, and whiskers indicate minimum and maximum values. The *P* values of the comparisons between PBS- and LPS-injected dams of each genotype were determined by Mann-Whitney *U* test. *P < 0.05; **P < 0.01; ***P < 0.001.

We then determined inflammasome activation in amniotic fluid following LPS injection (Figure 2A). First, using immunoblotting, we determined the activation of caspase-1 (CASP-1) and maturation of IL-1β in amniotic fluid. *Nlrp3*^–/–^ mice injected with LPS did not show increased activation of CASP-1 compared with PBS-injected controls, as *Nlrp3*^+/+^ mice did (Figure 2B). Consistently, KO mice had diminished levels of mature IL-1β compared with WT mice (Figure 2B). Therefore, *Nlrp3* deficiency impairs the activation of CASP-1 and subsequent processing and release of IL-1β in the amniotic cavity. Yet, the increased amniotic fluid concentrations of IL-1β in *Nlrp3*^–/–^ mice in response to LPS also indicate that microbial products activate alternative pathways leading to the processing and release of this proinflammatory cytokine (e.g., neutrophil elastase and cathepsin C; refs. 24, 34, 35).

Next, we characterized the intra-amniotic inflammatory response induced by LPS by profiling the concentrations of 36 proinflammatory cytokines in amniotic fluid. The classic markers of intra-amniotic inflammation, IL-6 and TNF (28, 36), were increased in response to LPS in *Nlrp3*^+/+^ and *Nlrp3*^–/–^ mice (Figure 2C). Similar results were observed for most measured cytokines (Supplemental Figure 2). Therefore, *Nlrp3*^+/+^ and *Nlrp3*^–/–^ mice display a similar intra-amniotic inflammatory response to microbial products. A possibility, however, was that such intra-amniotic inflammatory response exhibits differential NLRP3-dependent effects on the fetus. We profiled the expression of several inflammasome-associated and -independent inflammatory mediators in the fetal lung and intestine, tissues commonly affected by microbes invading the amniotic cavity (37, 38). LPS induced a strong inflammatory response in the fetal lung of both *Nlrp3*^+/+^ and *Nlrp3*^–/–^ mice (Figure 2, D–E, and Supplemental Figure 3) but not in the fetal intestine (Figure 2, F and G, and Supplemental Figure 4). However, key transcripts encoding proteins related to NF-κB and chemokine signaling, namely *Nfkb2* and *Ccl17*, were downregulated or unchanged in the fetal lung of *Nlrp3*^–/–^ mice upon LPS injection (Figure 2H).

Taken together, these results show that *Nlrp3* deficiency attenuates the deleterious effects of LPS by disrupting CASP-1 cleavage and the subsequent release of mature IL-1β in the amniotic cavity as well as by restricting the activation of specific inflammatory pathways in fetal organs exposed to amniotic fluid.

### Nlrp3 deficiency protects against preterm birth by impairing the common pathway of parturition.

Next, we sought to investigate whether *Nlrp3* deficiency protects against preterm birth by interfering with the common pathway of parturition, including the activation of the fetal membranes, uterine contractility, and cervical dilation (3, 25).

### Fetal membrane activation.

**Figure 3:**
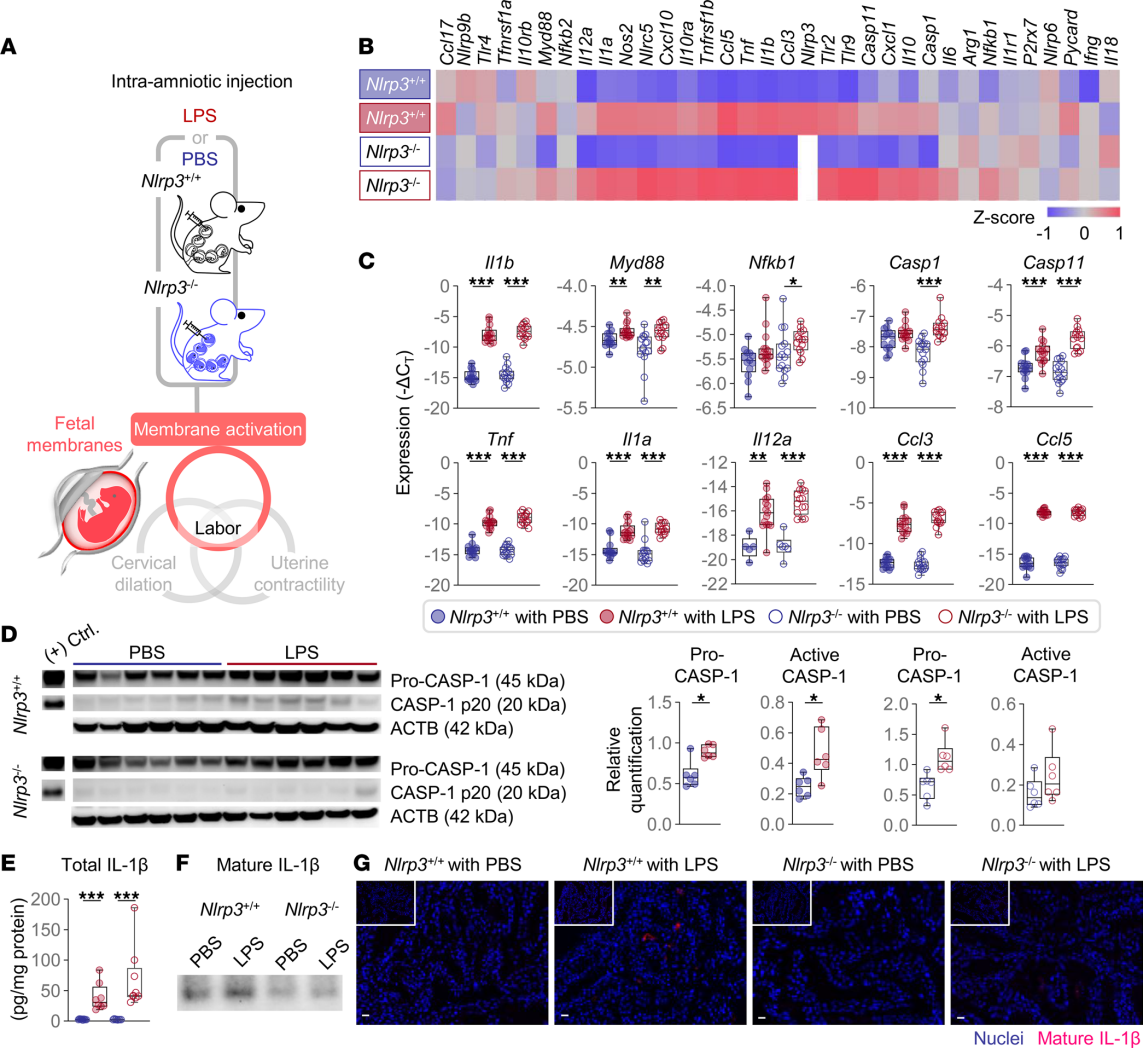
*Nlrp3* deficiency impairs LPS-induced fetal membrane activation. (**A**) Pregnant *Nlrp3*^+/+^ and *Nlrp3*^–/–^ mice were intra-amniotically injected with LPS (100 ng/25 μL) or PBS on 16.5 days post coitum (dpc). The fetal membranes were collected on 17.5 dpc. The spatial localization of the murine fetal membranes and a schematic diagram of the common pathway of labor are shown. (**B**) Heatmap visualization of inflammatory gene expression in the fetal membranes (*n* = 13–15 per group). (**C**) Expression of inflammation-associated genes in the fetal membranes (*n* = 13–15 per group). (**D**) Immunoblotting of pro-CASP and CASP-1 p20 in the fetal membranes (*n* = 6 per group). The expression was normalized by ACTB and shown as relative quantification. (**E**) Concentrations of total IL-1β in the fetal membranes (*n* = 8–9 per group). Values were adjusted by the total protein concentration in each sample. Data are shown as box-and-whisker plots where midlines indicate medians, boxes indicate interquartile ranges, and whiskers indicate minimum and maximum values. (**F**) Immunoblotting of mature IL-1β in fetal membrane lysates immunoprecipitated with anti–IL-1β antibody. Eight samples were pooled for each group. (**G**) Representative immunofluorescence images showing the expression of mature IL-1β in the fetal membranes (*n* = 3–4 per group). All images were taken at 200× original magnification. Scale bars represent 20 μm. Inset images show isotype control staining from the same case. The *P* values of the comparisons between PBS- and LPS-injected dams of each genotype were determined by Mann-Whitney *U* test. *P < 0.05; **P < 0.01; ***P < 0.001; (+) Ctrl., positive control.

Fetal membrane activation includes an inflammatory milieu characterized by the activation of the NF-κB pathway, leading to the synthesis of proinflammatory mediators as well as their processing machinery (e.g., the NLRP3 inflammasome) (39–41). Thus, we evaluated cytokine-driven inflammation in the fetal membranes as an indicator of tissue activation and profiled the expression of several inflammatory mediators (Figure 3A). The fetal membranes of both *Nlrp3*^+/+^ and *Nlrp3*^–/–^ mice displayed a strong upregulation of inflammation-associated genes upon LPS injection (Figure 3, B and C, and Supplemental Figure 5). Nonetheless, *Nlrp3*^–/–^ mice injected with LPS did not show increased CASP-1 activation compared to PBS-injected controls, as *Nlrp3*^+/+^ mice did (Figure 3D). Although the total IL-1β levels in the fetal membranes were increased in WT and KO mice in response to LPS (Figure 3E), immunoblotting and immunofluorescence consistently demonstrated that, in *Nlrp3*^–/–^ mice, the endotoxin did not increase the levels of mature IL-1β as it did in *Nlrp3*^+/+^ mice (Figure 3, F and G), consistent with the diminished activation of CASP-1. In addition, we observed that the expression of the apoptosis marker cleaved PARP-1 (42) did not colocalize with mature IL-1β in both WT and KO mice injected with LPS (Supplemental Figure 6), suggesting that inflammasome activation in the fetal membranes during LPS-induced intra-amniotic inflammation occurs independently of apoptotic cell death. Together, these results indicate that *Nlrp3* deficiency impairs LPS-induced fetal membrane activation by interfering with the processing of CASP-1 and subsequent release of mature IL-1β without significantly influencing the transcription of other inflammatory mediators.

LPS can also induce the noncanonical activation of the NLRP3 inflammasome, mediated by CASP-11 (CASP-4/5 in humans) (43, 44), and thus active forms of this caspase were also determined in the fetal membranes. Both *Nlrp3*^+/+^ and *Nlrp3*^–/–^ mice displayed increased active forms of CASP-11 in the fetal membranes following injection of LPS (Supplemental Figure 7A), confirming that cleavage of this caspase occurs independently of the NLRP3 sensor molecule (43, 44).

### Uterine activation and contractility.

**Figure 4:**
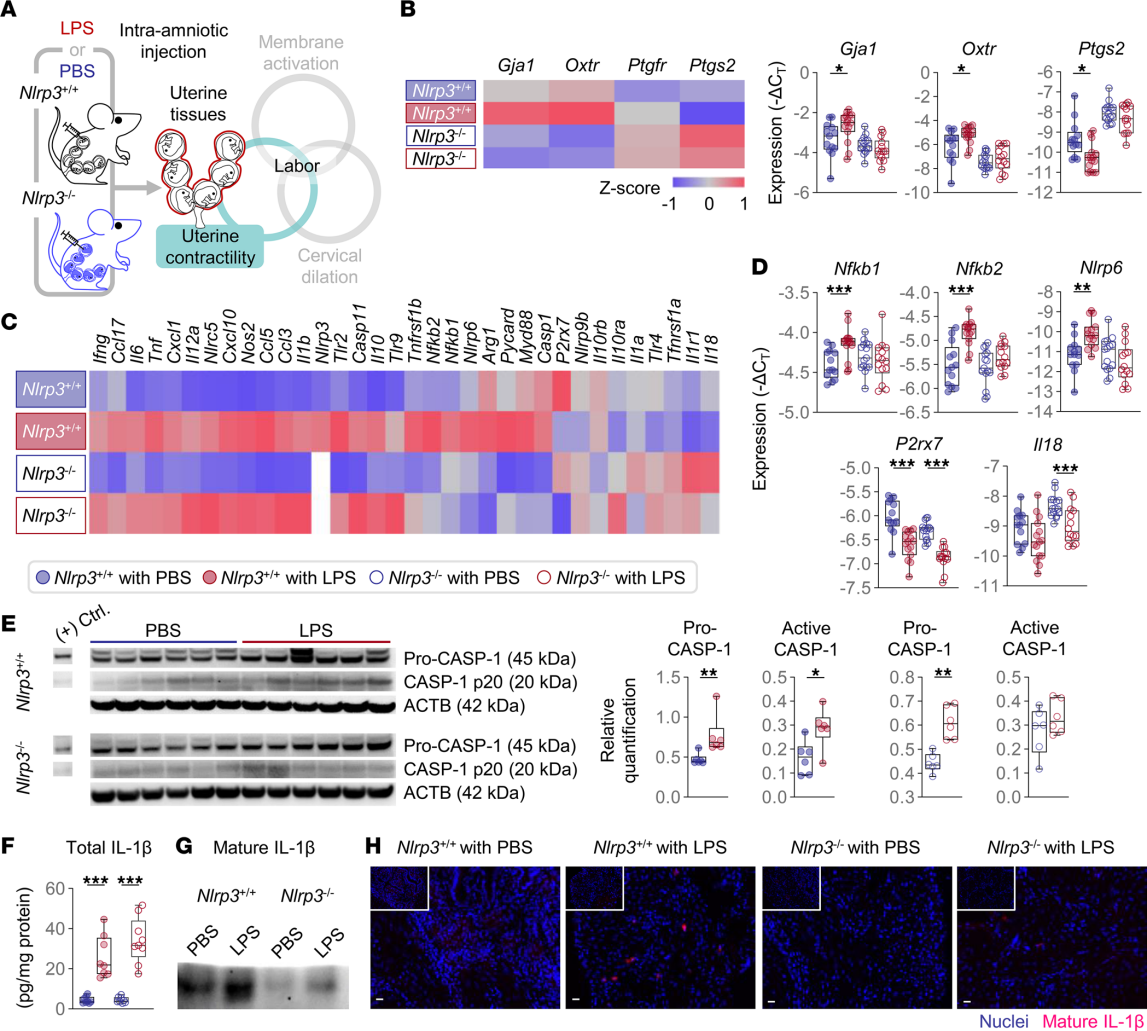
*Nlrp3* deficiency prevents LPS-induced uterine contractility and inflammation. (**A**) Pregnant *Nlrp3*^+/+^ and *Nlrp3*^–/–^ mice were intra-amniotically injected with LPS (100 ng/25 μL) or PBS on 16.5 days post coitum (dpc). The uterine tissues were collected on 17.5 dpc. The spatial localization of the murine uterus and a schematic diagram of the common pathway of labor are shown. (**B**) The expression of contractility genes in the uterus (*n* = 13–15 per group). (**C**) Heatmap visualization of the inflammatory gene expression in the uterus (*n* = 13–15 per group). (**D**) Expression of the inflammation-associated genes in the uterus (*n* = 13–15 per group). (**E**) Immunoblotting of pro-CASP and CASP-1 p20 in the uterus (*n* = 6 per group). The expression was normalized by ACTB and shown as relative quantification. (**F**) Concentrations of total IL-1β in the uterus (*n* = 8–9 per group). Values were adjusted by the total protein concentration in each sample. Data are shown as box-and-whisker plots where midlines indicate medians, boxes indicate interquartile ranges, and whiskers indicate minimum and maximum values. (**G**) Immunoblotting of mature IL-1β in uterine tissue lysates immunoprecipitated with anti-IL-1β antibody. Three samples were pooled in each group. (**H**) Representative immunofluorescence images showing the expression of mature IL-1β in the uterus (*n* = 6–10 per group). All images were taken at 200× original magnification. Scale bars represent 20 μm. Inset images show isotype control staining from the same case. The *P* values of the comparisons between PBS- and LPS-injected dams of each genotype were determined by Mann-Whitney *U* test. *P < 0.05; **P < 0.01; ***P < 0.001; (+) Ctrl., positive control.

The uterine tissues orchestrate the process of parturition as the transition from a quiescent to a contractile myometrium heralds the onset of labor (Figure 4A), which is accompanied by a surge of proinflammatory mediators (45, 46). This inflammatory milieu includes the activation of CASP-1 and release of mature IL-1β (47); however, the contribution of the NLRP3 inflammasome in the uterine tissues has yet to be established. First, we investigated the expression of contractility-associated genes as well as several inflammatory mediators in the uterine tissues following LPS injection (Figure 4A). Consistent with the onset of the active phase of labor, the uterine tissues of *Nlrp3*^+/+^ mice exhibited increased expression of transcripts encoding uterine activation proteins (*Gja1*, connexin-43; and *Oxtr*, oxytocin receptor) as well as downregulation of *Ptgs2* (COX-2) upon LPS injection (Figure 4B). Such phenomena were not observed in the uterine tissues of *Nlrp3*^–/–^ mice, suggesting impaired myometrial contractility. When exploring the expression of inflammasome-associated and -independent mediators, we found that the uterine tissues of *Nlrp3*^+/+^ and *Nlrp3*^–/–^ mice displayed upregulation of inflammation-associated genes upon LPS injection (Figure 4C and Supplemental Figure 8). Yet, the transcription of several key inflammation-associated molecules, such as *Nfkb1*, *Nfkb2*, *Nlrp6*, *P2rx7*, and *Il18*, was unchanged or downregulated in KO mice (Figure 4D). Next, we explored whether impaired uterine gene expression in *Nlrp3*^–/–^ mice was reflected by the reduced activation of CASP-1 and release of mature IL-1β. The uterine tissues of WT dams injected with LPS contained high quantities of active CASP-1, whereas those from *Nlrp3*^–/–^ mice did not (Figure 4E). Notably, although total IL-1β was increased in the uterine tissues of both WT and KO mice in response to LPS (Figure 4F), *Nlrp3*^–/–^ mice did not display a robust rise in the levels of mature IL-1β as *Nlrp3*^+/+^ mice did (Figure 4, G and H). The activation of CASP-11 in the uterine tissues upon LPS injection was similar between WT and KO mice (Supplemental Figure 7B). In sum, these data demonstrate, for the first time to our knowledge, a central role for NLRP3 inflammasome activation in the inflammatory processes that accompany myometrial contractility, an essential component of the common pathway of labor, in response to microbial products.

### Cervical dilation.

**Figure 5:**
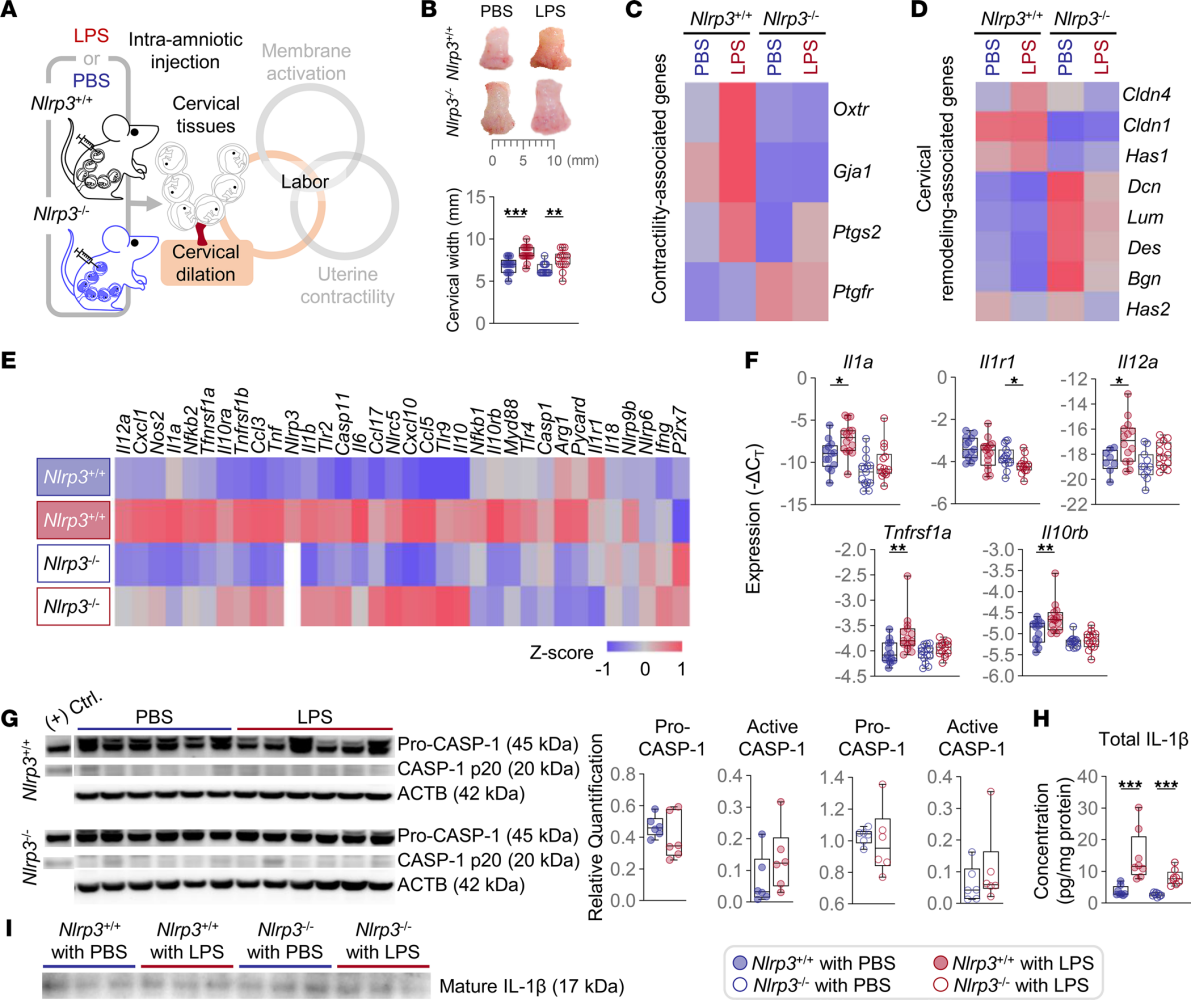
*Nlrp3* deficiency alters LPS-induced cervical dilation independently of inflammasome activation. (**A**) Pregnant *Nlrp3*^+/+^ and *Nlrp3*^–/–^ mice were intra-amniotically injected with LPS (100 ng/25 μL) or PBS on 16.5 days post coitum (dpc). The cervical tissues were collected on 17.5 dpc. The spatial localization of the murine cervix and a schematic diagram of the common pathway of labor are shown. (**B**) Representative images of the cervical dilation and width of the cervix (*n* = 13–15 per group). (**C**–**E**) Heatmap visualization of the (**C**) contractility-associated, (**D**) cervical remodeling–associated, and (**E**) inflammation-associated genes in the cervix (*n* = 10–15 per group). (**F**) Expression of inflammation-associated genes in the cervix (*n* = 13–15 per group). (**G**) Immunoblotting of pro-CASP and CASP-1 p20 in the cervix (*n* = 6 per group). The expression was normalized by ACTB and shown as relative quantification. (**H**) Concentrations of total IL-1β in the cervix (*n* = 8–9 per group). Values were adjusted by the total protein concentration in each sample. Data are shown as box-and-whisker plots where midlines indicate medians, boxes indicate interquartile ranges, and whiskers indicate minimum and maximum values. The *P* values of the comparisons between PBS- and LPS-injected dams of each genotype were determined by Mann-Whitney *U* test. (**I**) Immunoblotting of mature IL-1β in cervical tissue lysates immunoprecipitated with anti–IL-1β antibody (*n* = 3 per group). *P < 0.05; **P < 0.01; ***P < 0.001; (+) Ctrl., positive control.

The cervix is the gatekeeper of pregnancy since its dilation facilitates the delivery of the offspring (48, 49). Cervical dilation is an inflammatory process that includes structural changes to allow the distension of this organ during the process of labor (49, 50). Therefore, we also investigated whether *Nlrp3*^–/–^ mice display alterations in the inflammatory and contractility processes associated with cervical dilation (Figure 5A). *Nlrp3*^+/+^ mice displayed augmented distension of the cervix (Figure 5B) as well as increased expression of contractility genes (*Gja1*, *Oxtr*, and *Ptgs2*) in the cervical tissues upon LPS injection (Figure 5C and Supplemental Figure 9A). LPS also induced cervical distension in *Nlrp3*^–/–^ mice; however, the expression of *Gja1* and *Oxtr* was not upregulated (Figure 5, B and C, and Supplemental Figure 9A), indicating impaired cervical dilation. We also investigated the expression of genes associated with the structural reorganization of the cervix during parturition (i.e., cervical remodeling) (51, 52), including small leucine-rich proteoglycans (53) (an extracellular matrix component; ref. 54), tight junction proteins (51), and hyaluronidases (48, 55) (Figure 5D and Supplemental Figure 9B). Overall, the expression pattern of those genes differed between *Nlrp3*^+/+^ and *Nlrp3*^–/–^ mice (Figure 5D and Supplemental Figure 9B), suggesting that NLRP3 directly or indirectly modulates cervical composition. Moreover, the expression profile of several cervical inflammatory transcripts in response to LPS differed between *Nlrp3*^–/–^ and *Nlrp3*^+/+^ mice (Figure 5, E and F, and Supplemental Figure 9C). Indeed, the expression of several key inflammatory mediators, such as *Il1a*, *Il1r1*, *Il12a*, *Tnfrsf1a*, and *Il10rb*, was upregulated in WT mice injected with LPS but was unchanged or downregulated in KO mice (Figure 5F). Therefore, *Nlrp3* deficiency restricts LPS-induced distension and inflammation in the cervical tissues.

A previous study has shown that cervical dilation occurs in the absence of NLRP3 inflammasome activation prior to intra-uterine inflammation-induced preterm birth (56). To further explore the participation of the NLRP3 inflammasome in our animal model of intra-amniotic inflammation-induced preterm birth, we evaluated the activation of CASP-1 and release of IL-1β by the cervical tissues. Consistently, activation of CASP-1 was negligible in the cervical tissues of *Nlrp3*^+/+^ mice upon LPS injection, and such an effect was also observed in *Nlrp3*^–/–^ mice (Figure 5G). Total IL-1β was increased in response to LPS in the cervical tissues of both WT and KO mice (Figure 5H). However, the levels of the mature form of IL-1β in cervical tissue lysates were negligible in both WT and KO mice (Figure 5I). Active forms of CASP-11 were observed in the cervical tissues of both WT and KO mice following LPS administration (Supplemental Figure 7C). These data provide further evidence that the canonical activation of the NLRP3 inflammasome does not contribute to the inflammatory process associated with cervical dilation; yet, the deficiency of the NLRP3 sensor molecule impairs the transcription of contractility and inflammatory mediators in response to microbial products.

Collectively, this series of investigations shows a critical role for NLRP3 inflammasome activation in fetal membrane activation and uterine contractility, highlighting the fetal and maternal contributions of NLRP3 signaling to the common pathway of parturition leading to preterm birth. In addition, these results show that the NLRP3 molecule regulates inflammasome activation–independent processes associated with cervical dilation.

### Nlrp3 deficiency protects against preterm birth by limiting decidual inflammation.

**Figure 6:**
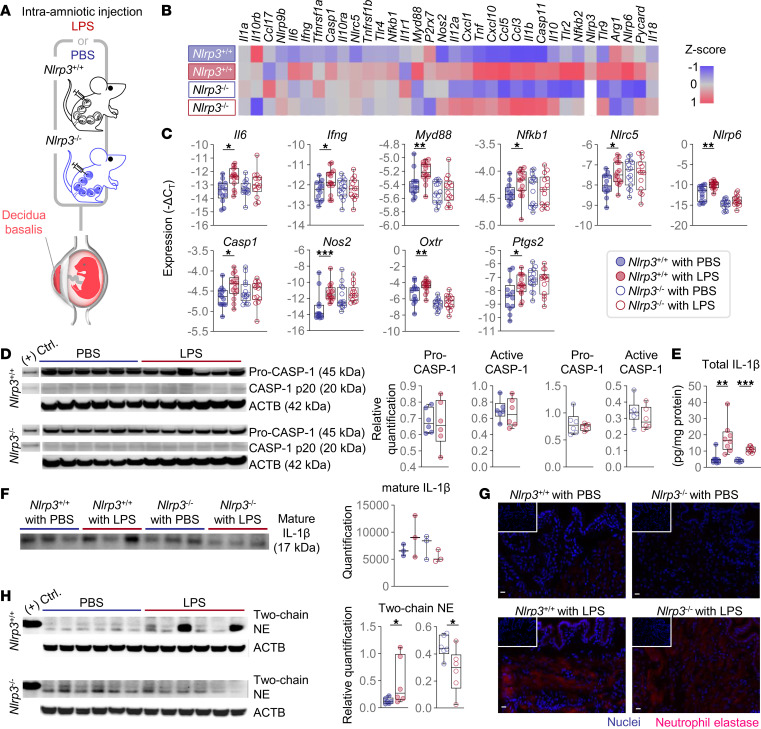
*Nlrp3* deficiency protects against preterm birth by limiting decidual inflammation. (**A**) Pregnant *Nlrp3*^+/+^ and *Nlrp3*^–/–^ mice were intra-amniotically injected with LPS (100 ng/25 μL) or PBS on 16.5 days post coitum (dpc). We collected the decidua basalis on 17.5 dpc. The spatial localization of the murine decidua is shown. (**B**) Heatmap visualization of inflammatory gene expression in the decidua (*n* = 13–15 per group). (**C**) Expression of the inflammation-associated genes (*n* = 13–15 per group). (**D**) Immunoblotting of pro-CASP and CASP-1 p20 in the decidua (*n* = 6 per group). The expression was normalized by ACTB and shown as relative quantification. (**E**) Concentrations of total IL-1β in the decidua. Values were adjusted by the total protein concentration in each sample (*n* = 8–9 per group). (**F**) Immunoblotting of mature IL-1β in decidual tissue lysates immunoprecipitated with anti–IL-1β antibody (*n* = 3 per group). (**G**) Representative immunofluorescence images showing the expression of neutrophil elastase (NE) in the decidua (*n* = 5–7 per group). All images were taken at 200× original magnification. Scale bars represent 20 μm. Inset images show isotype control staining from the same case. (**H**) Immunoblotting of 2-chain NE in decidual tissue lysates (*n* = 6 per group). Data are shown as box-and-whisker plots where midlines indicate medians, boxes indicate interquartile ranges, and whiskers indicate minimum and maximum values. The *P* values of the comparisons between PBS- and LPS-injected dams of each genotype were determined by Mann-Whitney *U* test. *P < 0.05; **P < 0.01; ***P < 0.001; (+) Ctrl., positive control.

The onset of labor is accompanied by a surge of proinflammatory mediators, including IL-1β, in the decidual tissues, a process that is exacerbated in the context of intra-amniotic inflammation (26, 57, 58). However, the role of NLRP3 inflammasome activation in such a process is unknown. Therefore, we next interrogated the expression of several inflammatory mediators in the decidua basalis following LPS administration (Figure 6A). The decidual tissues of both *Nlrp3*^+/+^ and *Nlrp3*^–/–^ mice displayed upregulation of inflammation-associated genes upon LPS injection (Figure 6B and Supplemental Figure 10A). Nonetheless, the expression of specific transcripts, namely *Il6*, *Ifng*, *Myd88*, *Nfkb1*, *Nlrc5*, *Nlrp6*, *Casp1*, *Nos2*, *Oxtr*, and *Ptgs2*, was upregulated in *Nlrp3*^+/+^ mice, but not in *Nlrp3*^–/–^, in response to LPS (Figure 6C). Notably, the active form of CASP-1 was negligible in the decidual tissues of WT and KO mice and did not change upon LPS injection (Figure 6D). The total levels of IL-1β were increased upon LPS injection in both *Nlrp3*^+/+^ and *Nlrp3*^–/–^ mice (Figure 6E); however, the mature form of this cytokine was not increased (Figure 6F). Given that decidual inflammation involves neutrophil recruitment (58, 59), and active CASP-1 was not found in the decidua of WT mice, we also investigated the presence of neutrophil elastase (NE) (an enzyme that promotes the processing of mature IL-1β, ref. 34). We observed that the protein expression of NE was increased in both LPS-injected *Nlrp3*^+/+^ and *Nlrp3*^–/–^ mice compared with PBS-injected controls (Figure 6G). NE can undergo self-cleavage from a single-chain to a 2-chain form with distinct catalytic activity (60); therefore, we performed immunoblotting to separate these forms and found that the protein expression of 2-chain NE was increased by LPS in *Nlrp3*^+/+^ mice but decreased in *Nlrp3*^–/–^ mice (Figure 6H). Conversely, single-chain NE was increased by LPS in both *Nlrp3*^+/+^ and *Nlrp3*^–/–^ mice (Supplemental Figure 10B). Last, the activation of CASP-11 in the decidua occurred upon LPS administration in both *Nlrp3*^+/+^ and *Nlrp3*^–/–^ mice (Supplemental Figure 7D).

These results show that decidual inflammation triggered by microbial products does not include the activation of the NLRP3 inflammasome, yet this sensor molecule regulates the transcription of key mediators implicated in the local inflammatory milieu that accompanies preterm labor and birth.

### Nlrp3 deficiency protects against preterm birth by dysregulating the functions of neutrophils and macrophages infiltrating the decidua and uterus.

**Figure 7:**
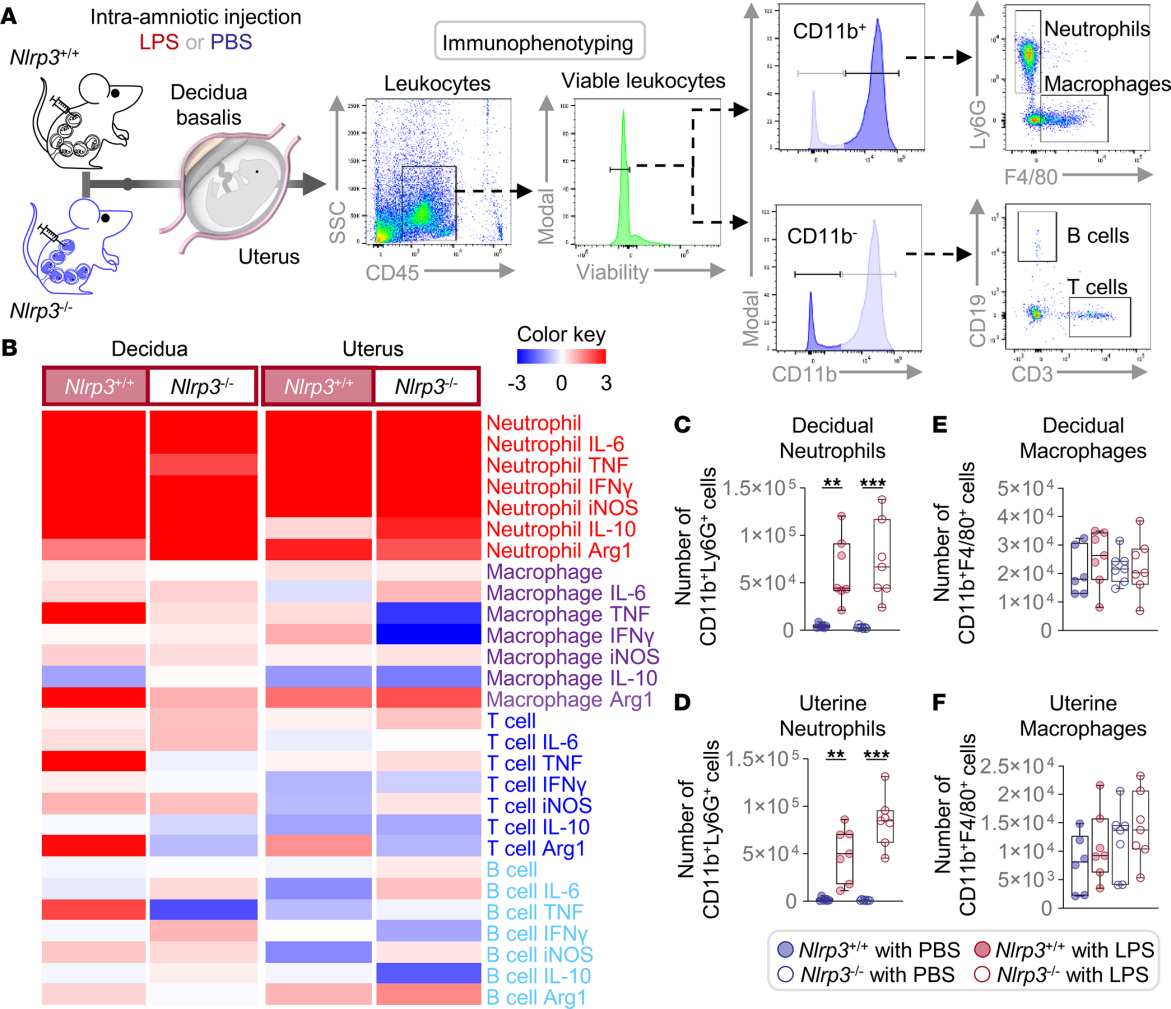
Immunophenotypes of infiltrating immune cells in the decidua and uterus. (**A**) Pregnant *Nlrp3*^+/+^ and *Nlrp3*^–/–^ mice were intra-amniotically injected with LPS (100 ng/25 μL) or PBS on 16.5 days post coitum (dpc) (*n* = 6–7 per group). The decidual and uterine tissues were collected on 17.5 dpc. A representative gating strategy used for immunophenotyping of decidual and uterine leukocytes is shown. (**B**) Heatmap visualization of changes in the log_2_-transformed numbers of immune cell subsets in the decidua and uterus. Red and blue indicate increased and reduced abundance, respectively, relative to the PBS controls of each genotype. (**C**–**F**) Numbers of macrophages and neutrophils in the (**C** and **E**) decidua and (**D** and **F**) uterus. Data are shown as box-and-whisker plots where midlines indicate medians, boxes indicate interquartile ranges, and whiskers indicate minimum and maximum values. The *P* values of the comparisons between PBS- and LPS-injected dams of each genotype were determined by Mann-Whitney *U* test. **P < 0.01; ***P < 0.001.

A critical maternal component of the inflammatory response, triggered by microbial products leading to preterm birth, is leukocyte infiltration of the reproductive tissues (61). Indeed, women with preterm labor and intra-amniotic inflammation display an influx of neutrophils and macrophages into the decidua (62) and myometrium (63), a phenomenon that is partially replicated in animal models (58, 64). Therefore, we next investigated whether the deficiency of *Nlrp3* alters neutrophil and macrophage infiltration and inflammatory mediator expression in response to LPS, as well as that of T and B cells, in the decidual and uterine tissues (Figure 7A). The immunophenotypes of such cells are displayed as a heatmap in Figure 7B. Neutrophil influx was markedly increased in the tissues of *Nlrp3*^+/+^ and *Nlrp3*^–/–^ mice in response to LPS; however, the response of both groups of mice was comparable (Figure 7, C and D). Decidual and uterine neutrophils in LPS-injected *Nlrp3*^+/+^ and *Nlrp3*^–/–^ mice displayed high levels of IL-6 and inducible NOS compared with controls (Supplemental Figure 11, A and B). By contrast, macrophage influx was unaltered upon LPS administration in both WT and KO mice (Figure 7, E and F), and their expression of inflammatory mediators was largely unaffected (Supplemental Figure 11, C and D). No differences in the infiltration of T and B cells were observed in the uterus or decidua of *Nlrp3*^+/+^ mice upon LPS administration (Supplemental Figure 12, A and D); however, these adaptive immune cells expressed high levels of TNF in the decidua (Supplemental Figure 12, B and C). Although T cell infiltration was greater in the decidua and uterus of *Nlrp3*^–/–^ mice injected with LPS compared with PBS controls (Supplemental Figure 12, A and D), their inflammatory mediator profiles did not differ (Supplemental Figure 12, B and E). No major phenotypic changes were observed for uterine B cells (Supplemental Figure 12F). These data indicate that the influx and phenotypes of innate and adaptive immune cells in the uterus and decidua are not drastically impaired by *Nlrp3* deficiency.

**Figure 8:**
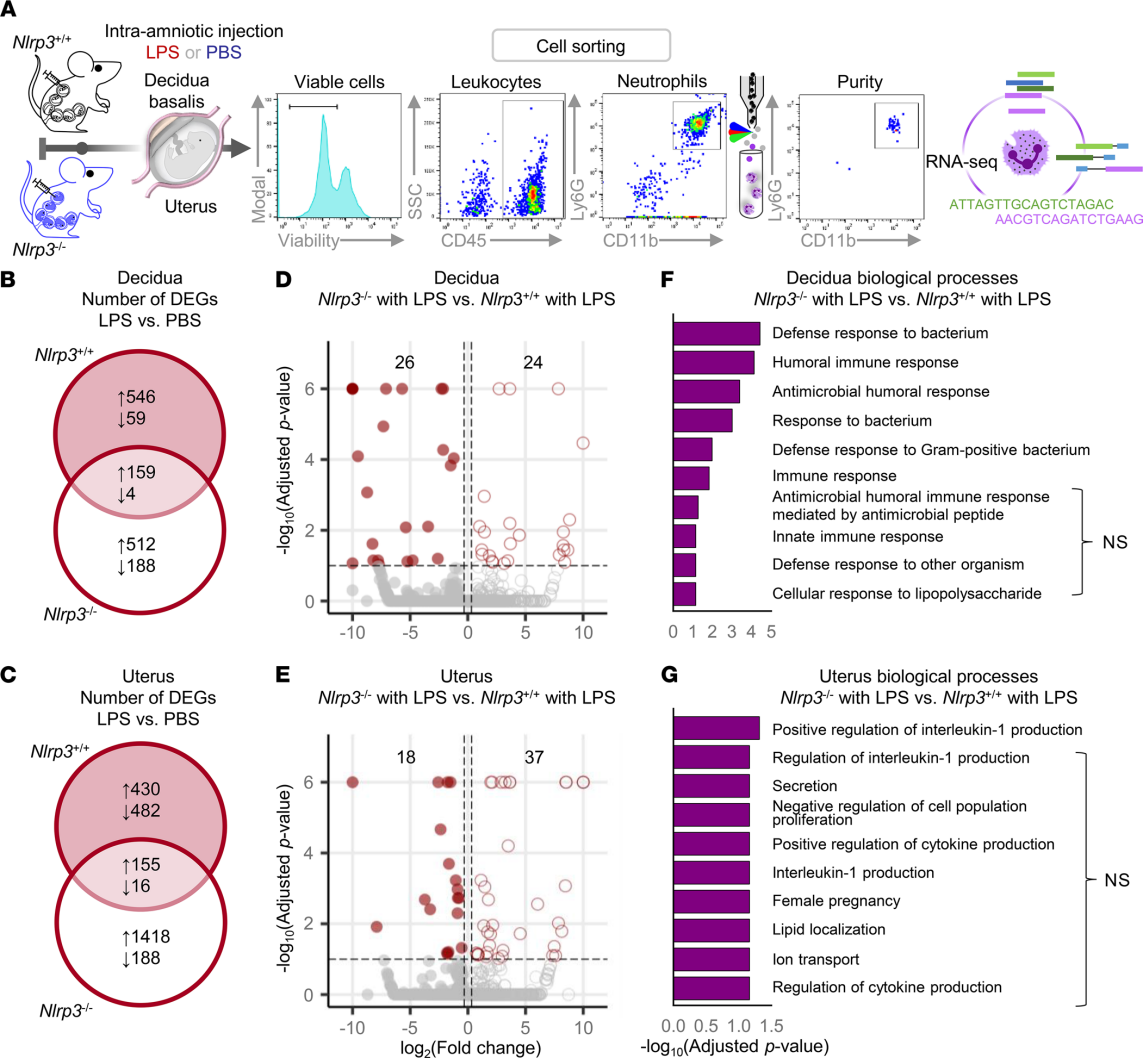
*Nlrp3* deficiency protects against preterm birth by dysregulating the functions of neutrophils infiltrating the decidua and uterus. (**A**) Pregnant *Nlrp3*^+/+^ and *Nlrp3*^–/–^ mice were intra-amniotically injected with LPS (100 ng/25 μL; *Nlrp3*^+/+^ dams, *n* = 8, *Nlrp3*^–/–^ dams, *n* = 7) or PBS (*Nlrp3*^+/+^ dams, *n* = 7, *Nlrp3*^–/–^ dams, *n* = 8) on 16.5 days post coitum (dpc). The decidual and uterine tissues were collected on 17.5 dpc. Neutrophils were sorted from these tissues by FACS. A representative gating strategy used for FACS of the decidual and uterine neutrophils is shown. To obtain sufficient quantities of RNA for RNA-Seq, 2 to 4 neutrophil samples (biological replicates) from each group were combined. Neutrophil transcriptomes were obtained by RNA-Seq (*n* = 2 per group). (**B** and **C**) Venn diagrams representing the overlap of DEGs in (**B**) decidual and (**C**) uterine neutrophils as determined by the comparison between LPS- and PBS-injected *Nlrp3*^–/–^ (unfilled circle) and *Nlrp3*^+/+^ (filled circle) dams. (**D** and **E**) Volcano plots showing the DEGs in (**D**) decidual and (**E**) uterine neutrophils from *Nlrp3*^–/–^ and *Nlrp3*^+/+^ dams injected with LPS. (**F** and **G**) Biological processes (BPs) enriched in the comparison between (**F**) decidual and (**G**) uterine neutrophils from *Nlrp3*^–/–^ and *Nlrp3*^+/+^ dams injected with LPS. Hypergeometric distribution was used to test for significance in the case of BPs. The *P* values of BPs were adjusted by FDR.

Neutrophils from *Nlrp3*^–/–^ mice seemed to display mild alterations in their inflammatory mediator expression profiles; therefore, we next evaluated their transcriptome using RNA-Seq to fully appreciate the effect of *Nlrp3* deficiency on their functional status (Figure 8A). Consistent with their central role in host defense, the transcriptomic activity of decidual and uterine neutrophils was upregulated in response to LPS in both *Nlrp3*^+/+^ and *Nlrp3*^–/–^ mice (Figure 8, B and C). Although several differentially expressed genes (DEGs) were shared between LPS-injected WT and KO mice, each genotype displayed unique DEGs (Figure 8, B and C, and Supplemental Tables 1–6). When comparing *Nlrp3*^–/–^ to *Nlrp3*^+/+^ mice injected with LPS, 50 and 55 transcripts were dysregulated in the decidual and uterine neutrophils, respectively (Figure 8, D and E, and Supplemental Tables 7 and 8). In decidual neutrophils, the dysregulated transcripts were involved in the following biological processes: immune response, defense response to bacterium, and antimicrobial humoral response (Figure 8F). In uterine neutrophils, the dysregulated transcripts were primarily involved in positive regulation of IL-1 production (Figure 8G). These data show that *Nlrp3* deficiency differentially influences the transcriptome of decidual and uterine neutrophils, which may translate to their altered host defense responses to microbial products.

**Figure 9:**
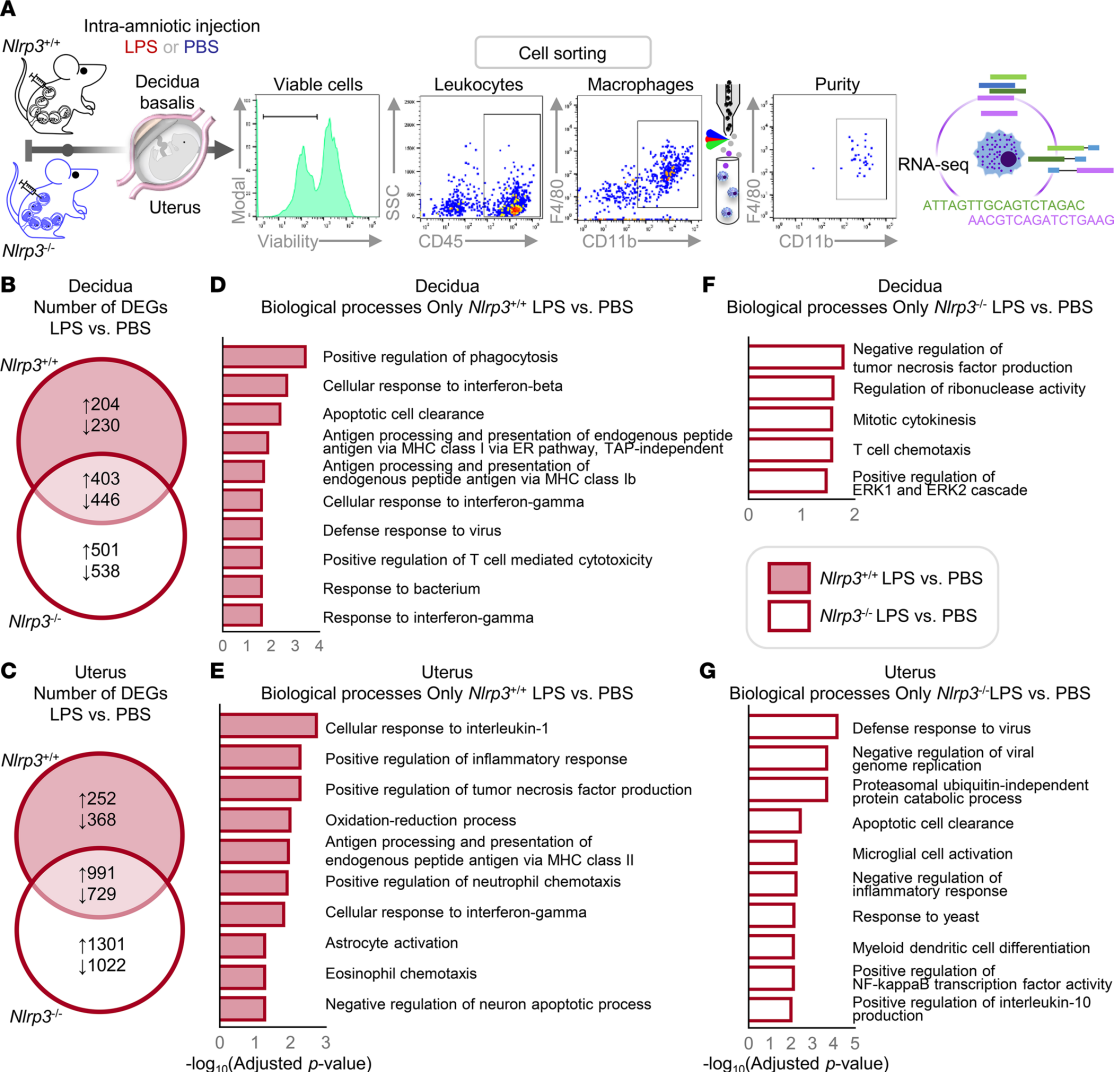
*Nlrp3* deficiency protects against preterm birth by dysregulating the functions of macrophages infiltrating the decidua and uterus. (**A**) Pregnant *Nlrp3*^+/+^ and *Nlrp3*^–/–^ mice were intra-amniotically injected with LPS (100 ng/25 μL) or PBS on 16.5 days post coitum (dpc) (*n* = 3 per group). The decidual and uterine tissues were collected on 17.5 dpc. Macrophages were sorted from these tissues by FACS. A representative gating strategy used for FACS of decidual and uterine macrophages is shown. Macrophage transcriptomes were obtained by RNA-Seq. (**B** and **C**) Venn diagrams representing the overlap of DEGs in the (**B**) decidual and (**C**) uterine macrophages as determined by the comparison between LPS- and PBS-injected *Nlrp3*^–/–^ (unfilled circle) and *Nlrp3*^+/+^ (filled circle) dams. (**D** and **E**) BPs enriched in the comparison between (**D**) decidual and (**E**) uterine macrophages from *Nlrp3*^+/+^ dams injected with LPS and those injected with PBS. (**F** and **G**) BPs enriched in the comparison between (**F**) decidual and (**G**) uterine macrophages from *Nlrp3*^–/–^ dams injected with LPS and those injected with PBS. Hypergeometric distribution was used to test for significance in the case of BPs. The *P* values of BPs were adjusted by high-specificity pruning.

Macrophages play a central role in innate host response against pathogens by performing phagocytosis (65) and, more importantly, serving as a source of proinflammatory cytokines such as IL-1β (66); hence, these innate immune cells possess the machinery required for its processing and release (67). Therefore, we performed RNA-Seq of sorted macrophages from the decidual and uterine tissues (Figure 9A). The transcriptomic activity of decidual and uterine macrophages was altered in response to LPS in *Nlrp3*^+/+^ and *Nlrp3*^–/–^ mice; yet, each genotype displayed unique DEGs (Figure 9, B and C, and Supplemental Tables 9–14). The biological processes enriched in decidual and uterine macrophages of WT mice in response to LPS were distinct from those of KO mice (Figure 9, D and E). Specifically, in *Nlrp3*^+/+^ mice, classical host response processes carried out by macrophages were observed, such as positive regulation of phagocytosis, cellular response to IL-1, and cellular response to interferons, among others (Figure 9, D and E). By contrast, in *Nlrp3*^–/–^ mice, such host response processes were absent, and instead, processes related to negative regulation of TNF production, defense against viruses, and T cell chemotaxis were observed in decidual and uterine macrophages (Figure 9, F and G). When comparing *Nlrp3*^+/+^ and *Nlrp3*^–/–^ mice injected with LPS, 155 and 173 transcripts were dysregulated in the decidual and uterine macrophages, respectively, of KO mice (Supplemental Figure 13, A and B, and Supplemental Tables 15 and 16). In decidual macrophages, the dysregulated transcripts were involved in the following biological processes: positive regulation of leukocyte differentiation, regulation of CD4^+^ T cell response, and leukocyte cell-cell adhesion, among others (Supplemental Figure 13C). In uterine macrophages, the dysregulated transcripts were involved in biological processes associated with cell migration and locomotion (Supplemental Figure 13D). These data demonstrate that *Nlrp3* deficiency alters the transcriptome of decidual and uterine macrophages, which may represent defects in classical macrophage functions, such as phagocytic activity and cellular regulation of IL-1 signaling.

Collectively, these data indicate that NLRP3 signaling profoundly affects neutrophil- and macrophage-mediated immune responses triggered by microbial products in the uterus and decidua without restraining their influx. Although NLRP3 signaling regulates host defense mechanisms in infiltrating neutrophils and macrophages in the decidua, it primarily modulates the manufacturing of IL-1β by these innate immune cells in the uterus, revealing tissue-specific functions of NLRP3 in the process of preterm labor.

### Nlrp3 deficiency improves uterine artery blood flow and constrains systemic cytokine responses in the mother.

**Figure 10:**
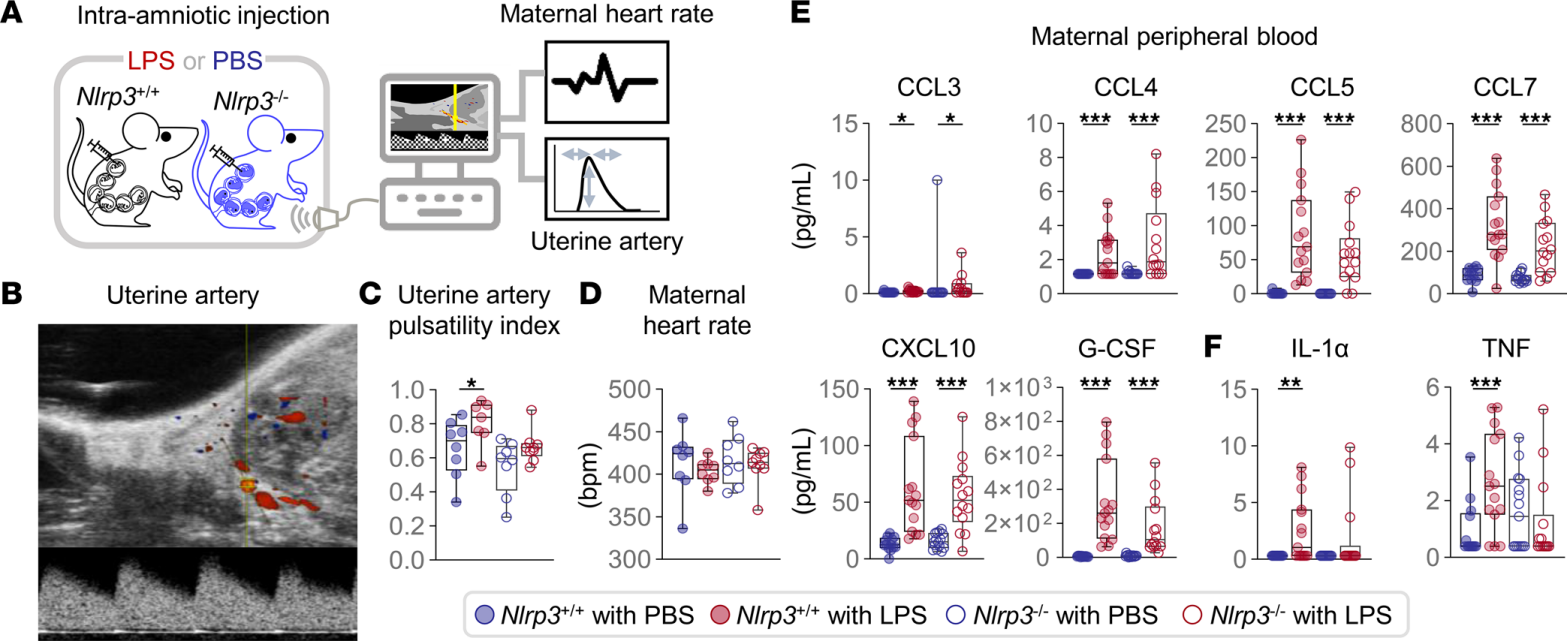
*Nlrp3* deficiency improves uterine artery blood flow and constrains systemic cytokine responses in the mother. (**A**) Pregnant *Nlrp3*^+/+^ and *Nlrp3*^–/–^ mice were intra-amniotically injected with LPS (100 ng/25 μL) or PBS on 16.5 days post coitum (dpc). Doppler ultrasonography was performed and the maternal plasma was collected on 17.5 dpc. (**B**) Representative Doppler ultrasound image of the uterine artery. (**C**) Uterine artery pulsatility index (*n* = 7–9 per group). (**D**) Maternal heart rate (*n* = 7–9 per group). (**E** and **F**) Maternal plasma concentrations of inflammatory mediators (*n* = 13–15 per group). Data are shown as box-and-whisker plots where midlines indicate medians, boxes indicate interquartile ranges, and whiskers indicate minimum and maximum values. The *P* values of the comparisons between PBS- and LPS-injected dams of each genotype were determined by Mann-Whitney *U* test. *P < 0.05; **P < 0.01; ***P < 0.001.

In addition to triggering local immune responses, intra-amniotic inflammation can also be reflected in maternal physiology (68). Indeed, pregnant women experiencing pregnancy complications associated with systemic inflammation display altered physiological parameters, such as an increased uterine artery pulsatility index (69). Therefore, we evaluated whether LPS-induced intra-amniotic inflammation alters maternal physiological parameters and whether *Nlrp3* deficiency could protect the mother against such alterations (Figure 10A). High-resolution Doppler ultrasound of the uterine artery (Figure 10B) revealed that, in *Nlrp3*^+/+^ mice, LPS caused an increase in its pulsatility index without altering maternal heart rate (Figure 10, C and D). Importantly, LPS injection did not cause alterations in the uterine artery pulsatility index in *Nlrp3*^–/–^ mice (Figure 10C). Moreover, *Nlrp3*^+/+^ mice injected with LPS displayed a mild systemic inflammatory response characterized by increased concentrations of specific chemokines and cytokines, including IL-1α and TNF (Figure 10, E and F, and Supplemental Figure 14), the latter being the prime mediator of the inflammatory response observed in septic shock (70). However, *Nlrp3*^–/–^ mice did not display a surge in IL-1α and TNF upon LPS administration (Figure 10F), Therefore, *Nlrp3* deficiency partially constrains systemic inflammation induced by microbial products while improving physiological parameters in the mother, providing further evidence of the maternal contribution to NLRP3 signaling implicated in the pathophysiology of preterm labor and birth.

### The fetal and maternal contribution of NLRP3 to the onset of preterm birth and subsequent neonatal mortality.

**Figure 11:**
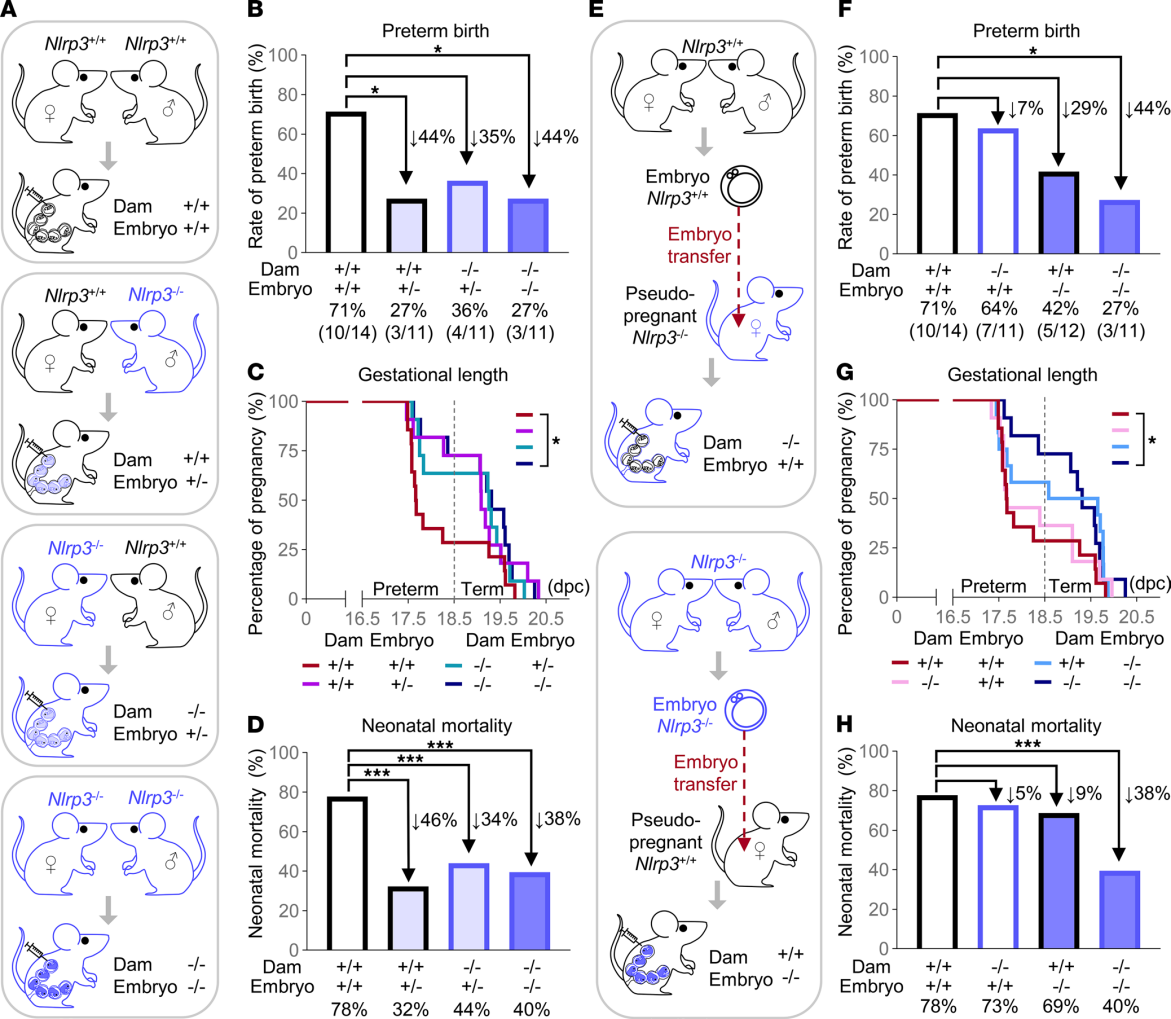
Fetal and maternal contribution of NLRP3 to the onset of preterm birth and subsequent neonatal mortality. (**A**) Mating strategies and resulting fetal genotypes. *Nlrp3*^+/+^ dams carrying *Nlrp3*^+/+^ fetuses, *Nlrp3*^+/+^ dams carrying *Nlrp3*^+/–^ fetuses, *Nlrp3*^–/–^ dams carrying *Nlrp3*^+/–^ fetuses, and *Nlrp3*^–/–^ dams carrying *Nlrp3*^–/–^ fetuses were injected with LPS (100 ng/25 μL) on 16.5 days post coitum (dpc) (*n* = 11–14 per group). (**B**) Preterm birth rates. (**C**) Gestational lengths. (**D**) Mortality rates of neonates. (**E**) Experimental design for embryo transfer. *Nlrp3*^+/+^ dams carrying *Nlrp3*^–/–^ fetuses and *Nlrp3*^–/–^ dams carrying *Nlrp3*^+/+^ fetuses were injected with LPS (100 ng/25 μL) on 16.5 dpc (*n* = 11–14 per group). (**F**) Preterm birth rates. (**G**) Gestational lengths. (**H**) Mortality rates of neonates. The *P* values for the rates of preterm birth and neonatal mortality were determined by Fisher’s exact test. The *P* values for the gestational lengths were determined by Gehan-Breslow-Wilcoxon test. *P < 0.05; ***P < 0.001.

Thus far, our data have shown that *Nlrp3* deficiency protects against preterm birth and neonatal mortality by disrupting inflammasome-dependent and -independent cellular and soluble immune responses in the fetal and maternal compartments, some of which are involved in the common pathway of parturition. Yet, whether fetal or maternal NLRP3 signaling governs such processes leading to preterm birth and neonatal mortality remained unclear. Therefore, we designed mating systems where *Nlrp3*^+/+^ and *Nlrp3*^–/–^ dams carry fetuses homozygous or heterozygous for *Nlrp3* (Figure 11A). As expected, *Nlrp3*^+/+^ dams carrying fetuses expressing this sensor molecule delivered preterm in 71% (10/14) of cases; however, only a fraction (27%, 3/11) of *Nlrp3*^+/+^ dams carrying heterozygous (*Nlrp3*^+/–^) fetuses delivered preterm (Figure 11B). Interestingly, only a fraction of *Nlrp3*^–/–^ dams carrying heterozygous (36%, 4/11) or homozygous (*Nlrp3*^–/–^) (27%, 3/11) fetuses delivered preterm (Figure 11B). These results were consistent with the shortened gestational length in WT dams carrying WT fetuses and extended gestation in the other experimental groups (Figure 11C). The observed rates of preterm birth were mirrored by the rates of neonatal mortality, in which a large proportion (78%) of WT neonates born to WT dams died shortly after delivery, whereas heterozygous neonates born to *Nlrp3*^+/+^ (32%) or *Nlrp3*^–/–^ (44%) dams were viable (Figure 11D). The latter result was similar in *Nlrp3*^–/–^ neonates born to *Nlrp3*^–/–^ dams (40%) (Figure 11D). Indeed, no differences in mortality were observed among heterozygous (*Nlrp3*^+/–^) neonates born to *Nlrp3*^+/+^ or *Nlrp3*^–/–^ dams (Figure 11D). To further appreciate the fetal or maternal contribution to LPS-induced adverse perinatal outcomes, embryo transfer experiments in which *Nlrp3*^+/+^ and *Nlrp3*^–/–^ dams carried either WT or KO embryos were performed (Figure 11E). Similar to WT dams carrying WT fetuses, a large proportion of *Nlrp3*^–/–^ dams carrying *Nlrp3*^+/+^ fetuses delivered preterm (64%, 7/11) (Figure 11F). On the other hand, only 42% (5/12) of *Nlrp3*^+/+^ dams carrying *Nlrp3*^–/–^ fetuses delivered preterm (Figure 11F). The rates of preterm birth were reflected by the gestational length (Figure 11G). Strikingly, *Nlrp3*^+/+^ or *Nlrp3*^–/–^ neonates born to KO or WT dams, respectively, were susceptible to LPS-induced mortality, which was similar to that displayed by *Nlrp3*^+/+^ neonates born to *Nlrp3*^+/+^ dams (Figure 11H). By contrast, *Nlrp3*^–/–^ neonates born to *Nlrp3*^–/–^ dams were protected against LPS-induced mortality (Figure 11H). These data demonstrate that both the fetus and the mother contribute to the NLRP3-mediated inflammatory processes leading to preterm birth and neonatal mortality upon microbial invasion of the amniotic cavity.

## Discussion

The amniotic cavity containing the fetus and the placenta is sterile (71). However, in some cases, bacteria from the lower genital tract can gain access to the amniotic cavity (5, 6), initiating local inflammatory responses without causing severe systemic inflammation in the mother (13). Thus, most cases of intra-amniotic inflammation associated with bacteria are subclinical in nature (3, 25, 29). Yet, it is well accepted that the localized intra-amniotic inflammatory response is sufficient to initiate the premature activation of the common pathway of labor leading to preterm delivery (3, 72). The animal model of preterm birth utilized herein resembles such a clinical scenario, since pregnant mice that receive ultrasound-guided intra-amniotic injection of LPS exhibit a localized inflammatory response in the amniotic cavity without a cytokine storm in the maternal circulation (27, 73). Using this model, we discovered that the majority of mice deficient for *Nlrp3* are protected against preterm birth and the subsequent mortality of premature neonates caused by intra-amniotic inflammation. Such findings emphasize the key role of NLRP3 inflammasome activation in the pathophysiology of preterm labor and birth. Therefore, we undertook a series of mechanistic investigations to decipher the biological processes regulated by NLRP3 signaling in the fetal and maternal compartments.

A major finding of our study was that both WT and *Nlrp3-*deficient mice injected with LPS displayed an intra-amniotic inflammatory response. Yet, *Nlrp3-*deficient mice displayed impaired inflammasome activation (i.e., activation of CASP-1 and maturation of IL-1β) in the amniotic fluid upon LPS injection. Previous studies have shown that the systemic or intra-amniotic administration of IL-1β alone induces preterm labor and birth in mice and nonhuman primates (72, 74), whereas administration of IL-1RA, the natural antagonist of the IL-1R, prevents preterm birth in mice (74). In addition, amniotic fluid concentrations of CASP-1 were increased in women who underwent spontaneous preterm labor with intra-amniotic inflammation compared with those without intra-amniotic inflammation (75). These results indicate that NLRP3 inflammasome activation and the subsequent maturation of IL-1β is a key process in the intra-amniotic cytokine storm that leads to preterm birth and that an inflammatory response lacking the activation of this signaling pathway is insufficient to facilitate the premature pathway of labor.

The common pathway of parturition is a well-orchestrated process that includes fetal membrane activation, uterine contractility, and cervical dilation (3, 25, 76). In the current study, we report that the intra-amniotic administration of LPS to *Nlrp3*-deficient mice triggered an inflammatory milieu in the fetal membranes similar to that observed in WT mice; however, the processing of mature IL-1β was defective as a consequence of impaired CASP-1 activation. Given that NLRP3 inflammasome activation occurs downstream of TLR4 signaling, it is reasonable to suggest that, while the expression of inflammatory genes is unaltered, the resulting activation of the fetal membranes is suppressed in *Nlrp3*^–/–^ mice. These data highlight a role for the NLRP3 inflammasome in the activation of the murine fetal membranes, consistent with previous studies showing that the chorioamniotic membranes of women with intra-amniotic inflammation display NLRP3 inflammasome priming and assembly in preterm (21, 22) and term (77) gestations. In vitro studies have also shown that the incubation of the chorioamniotic membranes with LPS results in the activation of CASP-1 (47), which can be specifically abrogated by a CASP-1 inhibitor (77). These results indicate that the NLRP3 inflammasome is required for the complete activation of the fetal membranes as part of the premature pathway of labor induced by microbes invading the amniotic cavity.

Myometrial contractility is a key process in the common pathway of labor that can be synergistically augmented by inflammation in the neighboring activated fetal membranes (78, 79). In the study herein, we provide the first evidence to our knowledge that NLRP3 inflammasome activation is essential for myometrial contractility, given that *Nlrp3-*deficient mice displayed reduced expression of uterine activation proteins and inflammatory mediators. Our data are in line with prior in vitro studies showing that incubation of human myometrial explants with LPS results in CASP-1 activation (47). Furthermore, our results provide an explanation as to why treatment with an inhibitor of NLRP3 inflammasome assembly (i.e., MCC950) suppresses premature uterine contractions induced by intra-amniotic inflammation (24, 80) and leads to the arrest of labor at term (80). Hence, the activation of the NLRP3 inflammasome is required for the coordinated contractility of the myometrium, the pacemaker of parturition (81), during intra-amniotic inflammation.

Herein, we report that the process of cervical dilation, which includes an inflammatory response, is halted in *Nlrp3-*deficient mice; yet, such events were not dependent on NLRP3 inflammasome activation. Given that uterine contraction and cervical dilation are codependent processes (82), the impaired uterine contractility observed in *Nlrp3*-deficient mice could disrupt cervical dilation. Moreover, we report dysregulated expression of genes associated with cervical remodeling in *Nlrp3*-deficient mice. These findings suggest that NLRP3 inflammasome activation is not required for cervical dilation; however, the NLRP3 sensor molecule may directly or indirectly participate in the inflammatory and distension-associated events in the cervix preceding preterm birth.

The decidua is the site of interaction between the mother and the fetal tissues (83) and harbors a local inflammatory milieu that includes cytokines/chemokines and infiltrating maternal immune cells (57, 61, 62, 84, 85) during physiological and pathological labor. Herein, we report that the NLRP3 sensor molecule contributes to such an inflammatory milieu. Furthermore, *Nlrp3* deficiency profoundly affected the transcriptome of neutrophils and macrophages in the decidua without impairing their LPS-induced influx. Specifically, our RNA-Seq data provide evidence that the NLRP3 molecule influences the host defense functions of decidual neutrophils, which we believe represents the first demonstration of a role for this sensor molecule in decidual neutrophil biology. Our RNA-Seq data also provide insight into the mechanisms implicated in decidual macrophage biology by demonstrating that the NLRP3 molecule regulates their functions in response to LPS. This concept is supported by previous reports indicating a critical role for the NLRP3 inflammasome in macrophage cytokine signaling (16, 86). Furthermore, the diminished functionality of decidual macrophages from *Nlrp3*-deficient mice contributes to the growing body of evidence suggesting that this sensor molecule plays a central role in macrophage biology (87). Notably, uterine neutrophils and macrophages displayed transcriptomic profiles distinct from those of their decidual counterparts, suggesting that the functionality of these innate immune cells is tailored to the specific uterine microenvironment and is regulated by the NLRP3 molecule.

Herein, we demonstrated that *Nlrp3* deficiency constrains the mild systemic inflammatory response induced by intra-amniotic LPS. Specifically, we report that the maternal systemic concentrations of IL-1β and TNF did not increase in *Nlrp3*-deficient mice injected with LPS. Both of these systemic cytokines have been implicated in the pathophysiology of preterm birth (18, 19, 36). The NLRP3 regulation of the maternal systemic inflammatory response has clinical relevance, given that women who ultimately deliver preterm display an elevated uterine artery pulsatility index (88), which was observed in WT mice injected with LPS and restored to physiological parameters in *Nlrp3*-deficient mice.

Notably, we also report that neonates born to *Nlrp3-*deficient mice injected with LPS delivered at term and thrived up to infancy, which was comparable to control mice. Yet, the intra-amniotic administration of LPS triggered acute inflammatory responses in both WT and KO mice, indicating divergent fetal consequences of such inflammation. In WT mice, the intra-amniotic response is sufficient to trigger preterm parturition and fetal compromise, which resembles the clinical sequelae (e.g., acute respiratory distress syndrome, ARDS; and bronchopulmonary dysplasia, BPD) observed in premature neonates born to women with microbial invasion of the amniotic cavity (89, 90). In KO mice, however, the acute intra-amniotic inflammatory response caused fetal bradycardia and the upregulation of several inflammatory mediators in the fetal lung without triggering preterm parturition. Therefore, these fetuses were maintained in utero, allowing for the resolution of hostile inflammation and proper fetal development. In addition, fetuses of *Nlrp3*-deficient mice showed suppressed activation of inflammatory pathways in the lung, which likely contributed to their survival. These data add to the current literature showing that the NLRP3 inflammasome participates in the mechanisms of tissue injury observed in neonatal morbidities, namely ARDS (91) and BPD (92). Hence, the NLRP3 inflammasome is implicated in pathological processes leading to fetal and neonatal disease.

Prior investigations indicated that the mechanisms responsible for preterm labor and birth fully rely on the mother (93, 94). Yet, there is solid evidence showing that the fetus plays a central role in the onset of labor (76, 95). In the current study, we clearly showed that both the fetus and the mother contribute to the mechanisms whereby NLRP3 signaling induces preterm labor and birth as well as adverse neonatal outcomes in the context of intra-amniotic inflammation.

It is worth mentioning that, besides the induction of inflammation through NLRP3 inflammasome activation, the NLRP3 molecule itself may play a role in the pathological processes associated with preterm birth and neonatal mortality caused by intra-amniotic inflammation. Indeed, we observed that cervical dilation and decidual inflammation were both tempered in *Nlrp3*-deficient mice independently of CASP-1 activation and maturation of IL-1β, and the baseline expression of cervical tissue genes was different between WT and KO mice. Consistently, the NLRP3 molecule has been previously reported as exhibiting inflammasome-independent functions that include tissue repair (96), Th2 cell differentiation (97), restriction of protective early neutrophil responses (98), and activity as a transcription factor (99); therefore, it is plausible that such NLRP3-mediated, inflammasome-independent processes could be participating in cervical dilation and decidual inflammation. Moreover, this may also explain why *Nlrp3-*deficient mice seemed to have low concentrations of total IL-1β in the cervical and decidual tissues upon LPS administration. Future mechanistic studies will further elucidate the inflammasome-independent functions of NLRP3 and investigate how this molecule can participate in labor-associated signaling pathways in the reproductive tissues.

The current study has some potential limitations. First, we did not evaluate the effects of *Nlrp3* deficiency on membrane rupture, which facilitates the process of parturition (25). Our model of ultrasound-guided intra-amniotic injection involves minimal injury to the fetal membranes, which may lead to leakage of amniotic fluid and could mimic membrane rupture. Indeed, the surgical creation of holes in the fetal membranes has been utilized as a model of membrane rupture in another study (100). However, in this study the control group also underwent intra-amniotic injection, and thus any secondary effects of the injection itself were controlled for. Moreover, neither *Nlrp3*^+/+^ nor *Nlrp3*^–/–^ dams intra-amniotically injected with PBS delivered preterm in this study, suggesting that the minimal fetal membrane injury resulting from ultrasound-guided intra-amniotic injection is insufficient to induce preterm birth. Second, in this study we utilized a syngeneic mating strategy, since NLRP3 inflammasome activation is primarily involved in the innate immune response (17). Indeed, we showed herein that the infiltration and function of adaptive immune cells at the materno-fetal interface was unaffected by NLRP3 deficiency. Yet, we cannot rule out potential differences in the NLRP3 signaling pathway between syngeneic and allogeneic pregnancies, and additional investigation will be required to address these points.

In conclusion, this study provides mechanistic evidence of a central role for the NLRP3 inflammasome in the fetal and maternal signaling required for the premature onset of labor leading to preterm birth. First, we report that NLRP3-mediated processing of mature IL-1β is necessary for the complete intra-amniotic inflammatory cascade that accompanies preterm labor and leads to fetal injury resulting in neonatal death. Next, we demonstrated that the NLRP3 pathway is essential for triggering the fetal and maternal mechanisms responsible for preterm parturition, including fetal membrane activation, uterine contractility, and cervical dilation as well as decidual inflammation. In addition, we found that, although NLRP3 is not implicated in intra-amniotic inflammation-induced leukocyte recruitment into the uterus and decidua, it profoundly regulates the RNA-Seq–inferred functional status of neutrophils and macrophages in this maternal compartment. Last, we revealed a dual contribution of NLRP3 signaling by the fetus and the mother to the premature activation of the labor cascade during intra-amniotic inflammation, highlighting the hitherto unappreciated fetal-maternal dialog in the syndrome of preterm birth. This study sheds light on the molecular underpinnings of intra-amniotic inflammation resulting in preterm labor and birth, the leading cause of neonatal morbidity and mortality worldwide.

## Methods

More information regarding the study design, laboratory procedures, and data analysis is in the Supplemental Methods.

### Data and materials availability.

Data generated in this study are included in the manuscript and/or in the supplemental materials. Generated RNA-Seq data were deposited in the NCBI’s Gene Expression Omnibus (accession number GSE183698).

### Study approval.

All procedures were approved by the Institutional Animal Care and Use Committee at Wayne State University under Protocol Nos. A-07-03-15, 18-03-0584, and 21-04-3506.

## Author contributions

KM performed experiments, analyzed data, and provided intellectual input. RR analyzed data and provided intellectual input. JG performed experiments and analyzed data. LT performed experiments and analyzed data. VGF performed experiments and analyzed data. YX performed experiments and analyzed data. BD analyzed data and provided intellectual input. MAH performed experiments and analyzed data. DM analyzed data and provided intellectual input. PGC analyzed data and provided intellectual input. MFJ analyzed data and provided intellectual input. SA analyzed data and provided intellectual input. LIG analyzed data and provided intellectual input. ALT analyzed data and provided intellectual input. NGL conceived and designed the study, analyzed data, and provided intellectual input. All authors revised and provided feedback for the final version of the manuscript.

## Supplementary Material

Supplemental data

Supplemental tables 1-16

## Version 1 08/22/2022

Electronic publication
