# Supplementary material for: Fetal and maternal NLRP3 signaling is required for preterm labor and birth
Source: JCI Insight. 2022 Aug 22;7(16):e158238. doi: 10.1172/jci.insight.158238 (PMC9462488; doi:10.1172/jci.insight.158238)
Supplement: Supplemental data [file jciinsight-7-158238-s201.pdf]

SUPPPLEMENTARY MATERIALS for

Fetal and Maternal NLRP3 Signaling is Required for Preterm Labor and Birth

Supplemental Materials and Methods

Supplemental Figure 1. Prenatal evaluation of *Nlrp3*$^{-/-}$ mice.

Supplemental Figure 2. Amniotic fluid concentrations of inflammatory mediators.

Supplemental Figure 3. Gene expression in the fetal lung.

Supplemental Figure 4. Gene expression in the fetal intestine.

Supplemental Figure 5. Gene expression in the fetal membranes.

Supplemental Figure 6. Localization of mature IL-1β and cleaved PARP-1 in the fetal membranes.

Supplemental Figure 7. Caspase-11 activation in the fetal and maternal tissues.

Supplemental Figure 8. Gene expression in the uterus.

Supplemental Figure 9. Gene expression in the cervix.

Supplemental Figure 10. Inflammatory mediator expression in the decidua.

Supplemental Figure 11. Inflammatory mediator expression by decidual and uterine neutrophils and macrophages.

Supplemental Figure 12. Inflammatory mediator expression by decidual and uterine T cells and B cells.

Supplemental Figure 13. Transcriptomic changes in decidual and uterine macrophages.

Supplemental Figure 14. Maternal plasma concentrations of inflammatory mediators.

Supplemental Table 17. Sensitivities for the Luminex assay-based measurements of immune mediators.

Supplemental Table 18. Taqman assays utilized for RT-qPCR.

Supplemental Table 19. Antibodies utilized for immunophenotyping and fluorescence-activated cell sorting.

Provided as separate files:

Supplemental Table 1. Differentially expressed genes unique to decidual neutrophils from *Nlrp3*$^{+/+}$ mice determined by the comparison between LPS- and PBS-injected dams.

Supplemental Table 2. Differentially expressed genes unique to decidual neutrophils from *Nlrp3*$^{-/-}$ mice determined by the comparison between LPS- and PBS-injected dams.

Supplemental Table 3. Differentially expressed genes shared between decidual neutrophils from *Nlrp3*$^{-/-}$ and *Nlrp3*$^{+/+}$ mice determined by the comparison between LPS- and PBS-injected dams.

Supplemental Table 4. Differentially expressed genes unique to uterine neutrophils from *Nlrp3*$^{+/+}$ mice determined by the comparison between LPS- and PBS-injected dams.

Supplemental Table 5. Differentially expressed genes unique to uterine neutrophils from *Nlrp3*$^{-/-}$ mice determined by the comparison between LPS- and PBS-injected dams.

Supplemental Table 6. Differentially expressed genes shared between uterine neutrophils from *Nlrp3*$^{-/-}$ and *Nlrp3*$^{+/+}$ mice determined by the comparison between LPS- and PBS-injected dams.

Supplemental Table 7. Differentially expressed genes between decidual neutrophils from *Nlrp3*$^{-/-}$ mice injected with LPS and *Nlrp3*$^{+/+}$ mice injected with LPS.

Supplemental Table 8. Differentially expressed genes between uterine neutrophils from *Nlrp3*$^{-/-}$ mice injected with LPS and *Nlrp3*$^{+/+}$ mice injected with LPS.

Supplemental Table 9. Differentially expressed genes unique to decidual macrophages from *Nlrp3*$^{+/+}$ mice determined by the comparison between LPS- and PBS-injected dams.

Supplemental Table 10. Differentially expressed genes unique to decidual macrophages from *Nlrp3*$^{-/-}$ mice determined by the comparison between LPS- and PBS-injected dams.

Supplemental Table 11. Differentially expressed genes shared between decidual macrophages from *Nlrp3*$^{-/-}$ and *Nlrp3*$^{+/+}$ mice determined by the comparison between LPS- and PBS-injected dams.

Supplemental Table 12. Differentially expressed genes unique to uterine macrophages from *Nlrp3*$^{+/+}$ mice determined by the comparison between LPS- and PBS-injected dams.

Supplemental Table 13. Differentially expressed genes unique to uterine macrophages from *Nlrp3*$^{-/-}$ mice determined by the comparison between LPS- and PBS-injected dams.

Supplemental Table 14. Differentially expressed genes shared between uterine macrophages from *Nlrp3*$^{-/-}$ and *Nlrp3*$^{+/+}$ mice determined by the comparison between LPS- and PBS-injected dams.

Supplemental Table 15. Differentially expressed genes between decidual macrophages from *Nlrp3*$^{-/-}$ mice injected with LPS and *Nlrp3*$^{+/+}$ mice injected with LPS.

Supplemental Table 16. Differentially expressed genes between uterine macrophages from *Nlrp3*$^{-/-}$ mice injected with LPS and *Nlrp3*$^{+/+}$ mice injected with LPS.

SUPPLEMENTAL MATERIALS AND METHODS

Mice

Female and male C57BL/6 (WT or *Nlrp3*$^{+/+}$) mice (JAX stock #000664) and female and male B6.129S6-*Nlrp3*^{tm1Bhk} (*Nlrp3*$^{-/-}$) mutant mice (JAX stock #021302) were purchased from The Jackson Laboratory (Bar Harbor, ME, USA) and housed in the animal care facility at the C.S. Mott Center for Human Growth and Development at Wayne State University (Detroit, MI, USA). All mice were kept under a circadian cycle (light:dark = 12:12 h). Eight- to twelve-week-old females were mated with males of proven fertility. Female mice were checked daily between 8:00 a.m. and 9:00 a.m. to investigate the appearance of a vaginal plug, which indicated 0.5 days *post coitum* (dpc). Females with a vaginal plug were then housed separately from the males, their weights were monitored daily, and a weight gain of ≥2 grams by 12.5 dpc confirmed pregnancy. All animals were randomly assigned to experimental or control groups prior to the following experiments.

Animal model of LPS-induced intra-amniotic inflammation

Ultrasound-guided intra-amniotic injection of LPS (Cat#L4391; *Escherichia coli* O111:B4; Sigma-Aldrich, St. Louis, MO, USA) was used for modeling microbial-associated intra-amniotic inflammation in mice, as previously described (1-3). Briefly, dams were anesthetized on 16.5 dpc by inhalation of 1.75-2% isoflurane (Fluriso™ (Isoflurane, USP) VetOne, Boise, ID, USA) and intra-amniotically injected with LPS at concentrations of 100 ng per 25 µL of sterile 1X phosphate-buffered saline (PBS; Fisher Scientific Bioreagents, Fair Lawn, NJ, USA or Life Technologies Limited, Pailey, UK) in each amniotic sac under ultrasound guidance using the Vevo® 2100 Imaging System

(VisualSonics Inc., Toronto, Ontario, Canada) with a 30-gauge needle (Becton Dickinson, Franklin Lakes, NJ, USA). Control dams were intra-amniotically injected with 25 μL of PBS. Following the intra-amniotic injections, mice were placed under a heat lamp until they regained full motor function, which typically occurred within 10 min after removal from anesthesia.

Video monitoring of pregnancy outcomes

Pregnancy parameters, including the rates of preterm birth and neonatal mortality, were recorded *via* video camera (Sony Corporation, Tokyo, Japan). Gestational length was calculated from the presence of the vaginal plug (0.5 dpc) until the detection of the first pup in the cage bedding. Preterm birth was defined as delivery occurring before 18.5 dpc, and its rate was represented by the percentage of females delivering preterm among the total number of mice. The rate of neonatal mortality was defined as the proportion of delivered pups found dead among the total number of pups.

Evaluation of placental morphology and maternal-fetal obstetrical parameters by ultrasonography

Nlrp3$^{+/+}$ or *Nlrp3*$^{-/-}$ dams were intra-amniotically injected on 16.5 dpc with 100 ng/25 μL of LPS or 25 μL of PBS in each amniotic sac, as described above. Sixteen h after injection, placental morphology and maternal-fetal obstetrical parameters were evaluated by ultrasonography using the Vevo 2100 Imaging System with a 55-MHz linear ultrasound probe (VisualSonics Inc.), as previously described (4-9). Briefly, after induction of anesthesia, the placental thickness, diameter, and area were measured (Supplemental

Figure 1A) (6). Maternal-fetal obstetrical parameters were evaluated using Doppler ultrasonography. The maternal heart rate and pulsatility index (PI) were examined in the left and right uterine arteries, and the mean values were calculated and reported. The fetal heart rate and the peak systolic velocity (PSV) of the umbilical arteries of three fetuses from each dam were evaluated, which included the fetus most proximal to the cervix in each uterine horn. The PI was calculated using the following formula: PI = (peak systolic velocity – end diastolic velocity)/mean velocity.

Sampling from dams intra-amniotically injected with LPS

Pregnant *Nlrp3*$^{+/+}$ or *Nlrp3*$^{-/-}$ mice were intra-amniotically injected with 100 ng/25 μL of LPS or 25 μL of PBS in each amniotic sac on 16.5 dpc, as described above. Mice were euthanized on 17.5 dpc (16 h after injection), after which the maternal blood was collected by cardiac puncture and placed into a 1.5 mL safe-lock Eppendorf tube with heparin (Sigma-Aldrich). Plasma was separated from the maternal peripheral blood by centrifugation at 800 x g for 10 min at 4°C and stored at −20°C until analysis. Amniotic fluid was collected from each amniotic sac and centrifuged at 1,300 x g for 5 min at 4°C, after which the resulting supernatants were stored at -20°C until the determination of cytokine/chemokine concentrations. Animal dissection to obtain the decidua basalis, uterus (including predominantly myometrial tissue), cervix, fetal membranes, fetal lung, and fetal intestine was performed. Fetal and placental pictures were taken during the tissue dissection and their weights were recorded. Tissues were snap-frozen in liquid nitrogen (for cytokine determinations), preserved in RNA*later* Stabilization Solution (Invitrogen by Thermo Fisher Scientific, Carlsbad, CA, USA) according to the

manufacturer's instructions (for RT-qPCR), or embedded in Tissue-Tek OCT Compound (Sakura Finetek USA, Torrance, CA, USA) and stored at -80°C (for immunofluorescence staining).

A different cohort of pregnant *Nlrp3*$^{+/+}$ or *Nlrp3*$^{-/-}$ mice was intra-amniotically injected with 100 ng/25 µL of LPS or 25 µL of PBS in each amniotic sac on 16.5 dpc. Mice were euthanized on 17.5 dpc, 16 h after injection and immediately following anesthesia, for the evaluation of placental morphology and maternal-fetal obstetrical parameters by ultrasonography. Animal dissection to collect amniotic fluid, decidua basalis, uterus, cervix, and fetal membranes was performed as described above, and these samples were used for the immunoblotting experiments.

Immunoblotting of amniotic fluid and murine tissues

Tissue lysates of fetal membranes, decidua, uterus, and cervix were prepared by mechanically homogenizing snap-frozen tissues in PBS containing a complete protease inhibitor cocktail (Cat#11836170001; Roche Applied Sciences, Mannheim, Germany). Lysates were centrifuged at 15,700 x g for 5 min at 4°C and the supernatants were stored at −80°C until use. Total protein concentrations of amniotic fluid and tissue lysates were determined using the Pierce BCA Protein Assay Kit (Cat#23225; Pierce Biotechnology, Thermo Fisher Scientific, Inc., Rockford, IL, USA) prior to immunoblotting. Cell lysates and culture supernatants derived from murine bone marrow-derived macrophages (BMDMs) were utilized as positive controls for the expression of pro-CASP-1, CASP-1 p-20, pro-CASP-11, active CASP-11, and mature IL-1β, as previously described (3, 10, 11). Briefly, bone marrow was collected from female C57BL/6 mice and the cells were

differentiated in IMDM medium (Thermo Fisher Scientific) with 10% FBS (Invitrogen by Thermo Fisher Scientific) and 10 ng/mL of M-CSF (Cat#576402; BioLegend, San Diego, CA, USA) at 37°C and 5% CO_2 for 7 days. Resulting BMDMs were seeded into 6-well tissue culture plates (Fisher Scientific) at 5 × 10^5 cells/well and cultured at 37°C with 5% CO_2 overnight. The cells were then incubated with 0.5 µg/mL of LPS alone for 4 h followed by the addition of 10 µM of Nigericin (Cat# N7142, Sigma-Aldrich) for an additional 1 h to be used as positive controls for caspase-1, IL-1β, and neutrophil elastase or incubated with 50 µg/mL of *E. coli* outer membrane vesicles (OMVs; Cat#tlrl-omv; Invivogen, San Diego, CA, USA) for 6 h to be used as positive control for caspase-11. From the LPS- and Nigericin-treated BMDMs, the supernatants were collected and centrifuged at 1,300 x g for 5 min to remove floating cells and debris. The cell-free supernatants were then concentrated to 10X with the Amicon Ultra Centrifuge filter (Cat#UFC800324; Ultracel 3K, EMD Millipore, Darmstadt, Germany) and stored at −20°C until use. Cultured BMDMs were then collected and lysed with RIPA buffer (Cat#R0278; Sigma-Aldrich) containing a complete protease inhibitor cocktail. Lysates were centrifuged at 15,700 x g for 5 min at 4°C and the supernatants were collected and stored at −20°C until use. For the *E. coli* OMVs-treated BMDMs, cells were first lysed with 0.005% Digitonin (Cat#BN2006; Sigma-Aldrich) and the cytosol fraction was collected by centrifugation at 2,000 x g for 5 min. Representative tissue samples of the fetal membranes, uterus, cervix, and decidua from *Nlrp3*$^{+/+}$ mice intra-amniotically injected with LPS were run in a separate gel with the positive control for CASP-11 to confirm the tissue expression of pro-CASP-11 and CASP-11 p30 (Supplemental Figure 7E).

Amniotic fluid (27 µg total protein per well), tissue lysates from fetal membranes, decidua, uterus, and cervix (50-75 µg per well), and concentrated BMDM cell supernatants (20 µg) were subjected to electrophoresis in 4%–12% sodium dodecyl sulphate-polyacrylamide gels (Cat#NP0336BOX; Invitrogen). Separated proteins were then transferred onto nitrocellulose membranes (Cat#1620145; Bio-Rad, Hercules, CA, USA). Next, the nitrocellulose membranes were submerged in blocking solution (Cat#37542; StartingBlock T20 Blocking Buffer, Thermo Fisher Scientific) for 30 min at room temperature and then probed overnight at 4°C with the following antibodies: anti-mouse CASP-1 (Cat#14–9832-82; Thermo Fisher Scientific), anti-mouse neutrophil elastase (Cat# PA5-115648; Thermo Fisher Scientific), or (for amniotic fluid samples only) anti-mouse cleaved IL-1β (Cat#63124S; Cell Signaling Technology, Danvers, MA, USA). The membranes with tissue lysates from fetal membranes, decidua, uterus, and cervix were stripped with Restore PLUS Western Blot Stripping Buffer (Cat#46430; Thermo Fisher Scientific) for 15 min, washed with PBS, blocked, and re-probed overnight at room temperature with a rat anti-mouse CASP-11 antibody (Cat#14-9935-82; Thermo Fisher Scientific). Finally, nitrocellulose membranes with tissue lysates were washed with PBS, blocked, and re-probed for 1 h at room temperature with a mouse anti-β-actin (ACTB) monoclonal antibody (Cat#A5441, Sigma-Aldrich). After incubation with each primary antibody, membranes were incubated with horseradish peroxidase (HRP)-conjugated anti-rat IgG (Cat# 7077S, Cell Signaling; for CASP-1 and CASP-11), HRP-conjugated anti-rabbit IgG (Cat# 7074S, Cell Signaling; for mature IL-1β and neutrophil elastase), or HRP-conjugated anti-rabbit IgG (Cat# 7076S, Cell Signaling; for ACTB) for 1 h at room temperature. Chemiluminescence signals were detected with the ChemiGlow West

Chemiluminescence Substrate Kit (Cat#60-12596-00; ProteinSimple, San Jose, CA, USA) and images were acquired using the ChemiDoc Imaging System (Bio-Rad). Quantification was performed with ImageJ software (12). Briefly, each individual protein band on the blot image was automatically quantified by the software. The target protein expression in each individual sample of the fetal membranes, decidua, uterus, and cervix was normalized using the internal control, β-actin, in the same sample to obtain relative quantification. Internal controls were not utilized for amniotic fluid samples; yet, identical protein amounts were loaded for each sample.

Determination of cytokine concentrations in amniotic fluid and maternal plasma

The concentrations of amniotic fluid and maternal plasma cytokines/chemokines were determined using the ProcartaPlex Mouse Cytokine & Chemokine Panel 1A 36-plex (Invitrogen by Thermo Fisher Scientific), according to the manufacturer's instruction. This assay measures IFNα, IFNγ, IL-12p70, IL-1β, IL-2, TNF, GM-CSF, IL-18, IL-17A, IL-22, IL-23, IL-27, IL-9, IL-15/IL-15R, IL-13, IL-4, IL-5, IL-6, IL-10, Eotaxin (CCL11), IL-28, IL-3, LIF, IL-1α, IL-31, GRO-α (CXCL1), MIP-1α (CCL3), IP-10 (CXCL10), MCP-1 (CCL2), MCP-3 (CCL7), MIP- 1β (CCL4), MIP-2 (CXCL2), RANTES (CCL5), G-CSF, M-CSF, and ENA-78 (CXCL5). Plates were read using the Luminex FLEXMAP 3D (Luminex Corporation, Austin, TX, USA) and analyte concentrations were calculated using the xPONENT version 4.2 (Luminex). The sensitivities of the assays are shown in Supplemental Table 17.

RNA isolation, cDNA synthesis, and reverse transcription quantitative polymerase chain reaction analysis of murine tissues

Total RNA was isolated from the fetal membranes, uterus, cervix, decidua, fetal lung, and fetal intestine using QIAshredders (Qiagen, Germantown, MD, USA), RNase-Free DNase Sets (Qiagen) and RNeasy Mini Kits (Qiagen), according to the manufacturer's instructions. RNA concentrations, purity, and integrity were evaluated with the NanoDrop 8000 spectrophotometer (Thermo Scientific, Wilmington, DE, USA) and the Bioanalyzer 2100 (Agilent Technologies, Wilmington, DE, USA). cDNA was synthesized using SuperScript IV VILO Master Mix (Invitrogen by Thermo Fisher Scientific Baltics UAB, Vilnius, Lithuania). Gene expression profiling of the tissues was performed on the BioMark System for high-throughput RT-qPCR (Fluidigm, San Francisco, CA, USA) or the ABI 7500 Fast real-time PCR system (Applied Biosystems, Life Technologies Corporation, Pleasanton, CA, USA) with the TaqMan gene expression assays (Applied Biosystems, Life Technologies Corporation) listed in Supplemental Table 18.

Negative delta cycle threshold ($-\Delta C_T$) values were determined using multiple reference genes (*Gusb*, *Hsp90ab1*, *Gapdh*, and *Actb*) averaged within each sample for contractility-associated and inflammatory genes or *Gapdh* for cervical remodeling-associated genes. The $-\Delta C_T$ values were normalized by calculating the Z-score of each gene with all the groups (*Nlrp3*$^{+/+}$ dams injected with PBS, *Nlrp3*$^{+/+}$ dams injected with LPS, *Nlrp3*$^{-/-}$ dams injected with PBS, and *Nlrp3*$^{-/-}$ dams injected with LPS). Heatmaps were then created, which represent the mean of the Z-score of $-\Delta C_T$ and hierarchical

clustering with un-centered correlation using Subio Platform v1.24.5839 (Subio Inc., Kagoshima, Japan, https://www.subioplatform.com/).

Immunoassay determination of IL-1β concentrations in the fetal membranes, uterus, cervix, and decidua

Tissue lysates of the fetal membranes, uterus, cervix, and decidua were extracted by mechanical homogenizing of snap-frozen tissues in Cell Lysis Buffer 2 (Cat#895347; R&D Systems) for IL-1β quantification. Lysates were centrifuged at 15,700 x g for 5 min at 4°C and the supernatants were stored at −80°C until analysis. Total protein concentrations of tissue lysates were determined using the Pierce BCA Protein Assay Kit. The concentration of IL-1β in the tissue lysates was determined using the Mouse IL-1 beta/IL-1F2 Quantikine ELISA Kit (Cat#MLB00C; R&D Systems) following the manufacturer’s instructions. The sensitivity of the assay was 2.31 pg/mL. The concentrations of IL-1β were normalized with the total protein amounts in the tissue lysates.

Detection of mature IL-1β and neutrophil elastase in murine tissues by immunofluorescence staining

Tissue sections of 10 μm were obtained from fetal membrane, uterine, and decidual tissues preserved in Tissue-Tek OCT Compound and mounted on microscope slides. The slides were kept in -80°C until use. After warming up the slides to room temperature and rinsing with PBS, 4% paraformaldehyde (Electron Microscopy Sciences Hatfield, PA, USA) was used to fix the tissue sections for 15 min at room temperature.

Next, the tissue sections were permeabilized using 0.25% Triton X-100 (EMD Millipore, Billerica, MA, USA) for 5 min at room temperature. After rinsing with PBS, the slides were blocked with BlockAid Blocking solution (Cat# B10710; Thermo Fisher Scientific) for 1 h at room temperature. Then, the slides of the fetal membranes and uterus were incubated with rabbit anti-mouse cleaved-IL-1β antibody (Cat# 63124S; Cell Signaling Technology) or rabbit (DA1E) mAb IgG XP Isotype Control (Cell Signaling Technology) overnight at 4°C. The slides of the decidua were incubated with rabbit anti-mouse neutrophil elastase antibody (Cat# PA5-115648; Thermo Fisher Scientific) or rabbit immunoglobulin fraction (Cat# X0903, Dako/Agilent, Santa Clara, CA, USA) overnight at 4°C. The slides were washed three times with PBS containing 0.1% Tween 20 (PBS-T) (MP Biomedicals, LLC, Solon, OH, USA) for 10 min each. Next, slides were incubated with the secondary goat anti-rabbit IgG–Alexa Fluor 594 (Thermo Fisher Scientific) for 60 min at room temperature followed by three washes with PBS-T. Finally, slides were mounted using ProLong Diamond Antifade Mountant with DAPI (Thermo Fisher Scientific). Representative images were taken with the BZ-X810 Fluorescence Microscope (Keyence, Osaka, Japan) at 200X magnification.

Immunoprecipitation of mature IL-1β in murine tissues

Immunoprecipitation of cleaved IL-1β from fetal membrane, uterine, cervical, and decidual tissue lysates was performed using the Pierce™ Classic IP Kit (Cat# 26146; Thermo Fisher Scientific) following the manufacturer's instructions. Briefly, tissue lysates of the fetal membranes, uterus, cervix, and decidua were prepared by mechanically homogenizing snap-frozen tissues in IP lysis buffer containing a complete protease

inhibitor cocktail. Lysates were centrifuged at 13,000 x g for 10 min and the supernatants were collected. Culture supernatant derived from murine BMDMs was utilized as positive control. For the fetal membranes and uterine tissues, 8 and 3 samples, respectively, were pooled to obtain sufficient mature IL-1β protein signal. Total protein concentrations of tissue lysates were determined using the Pierce BCA Protein Assay Kit. Each lysate (1,000 µg for the fetal membranes, uterus, and decidua or 500 µg for the cervix) was pre-cleared using the control agarose resin and incubated with rabbit anti-mouse cleaved IL-1β antibody overnight at 4°C to form the immune complex. Next, the immune complex was captured using Pierce Protein A/G Agarose. After several washes to remove non-bound proteins, the immune complex was eluted with sample buffer and subjected to electrophoresis in 4%–12% sodium dodecyl sulphate-polyacrylamide gels and western blot transfer as described above. The blot was then incubated with rabbit anti-mouse cleaved IL-1β antibody followed by incubation with an HRP-conjugated anti-heavy chain of rabbit IgG antibody (Cat#HRP-66467, Proteintech, Rosemont, IL, USA). Chemiluminescence signals were detected with the ChemiGlow West Substrate Kit and images were acquired using the ChemiDoc Chemiluminescence Imaging System.

Localization of mature IL-1β and cleaved PARP-1

Nlrp3$^{+/+}$ dams were intra-amniotically injected with 100 ng/25 µL of LPS in each amniotic sac on 16.5 dpc, and euthanized 16 h post injection. The fetal membrane tissues were collected and fixed with 10% Neutral Buffered Formalin (Surgipath, Leyca Biosystems, Wetzlar, Germany). Tissues were paraffin-embedded and tissue sections (5-µm-thickness) were prepared. Multiplex immunofluorescence staining was performed

using the Opal Multiplex 7-color IHC kit (Cat#NEL811001KT; Akoya Biosciences, Marlborough, MA), according to the manufacturer's instructions. Each analyte was individually optimized with single antibody staining combined with different fluorescent TSA® reagents (Akoya Biosciences) prior to multiplex immunofluorescence staining. After deparaffinization and rehydration, slides were placed in antigen retrieval (AR) buffer and boiled using a microwave oven. Following blocking to eliminate nonspecific binding, slides were incubated with antibodies against mouse cleaved-IL-1β antibody (Cat#63124S; Cell Signaling Technology) or cleaved PARP-1 (Cat#44-698G; ThermoFisher Scientific) at room temperature. Then, the slides were washed and incubated with Opal Polymer anti-Rabbit HRP (Cat#ARH-2001EA; Akoya Biosciences). Next, the slides were incubated with one of the following fluorescent TSA® reagents included in the Opal 7-color IHC kit to detect each antibody staining: Opal 570 or Opal 690 (1:100 dilution). After washing, the slides were counterstained with Spectral DAPI (Cat#FP1490; Akoya Biosciences) and mounted using ProLong Diamond Antifade Mountant (Life Technologies, Eugene, OR). Autofluorescence slides as well as slides stained with isotype (negative controls) were included. Multiplex staining was performed by consecutively staining slide-mounted tissues using the same antibody concentrations and conditions validated through single-plex staining. Each previous primary and secondary antibody was removed by boiling in AR buffer before the application of the next primary antibody. After multiplex staining, the slides were imaged using the Vectra Polaris Multispectral Imaging System (Akoya Biosciences) at 40X magnification. BMDMs (prepared as mentioned above) were seeded on cover glasses overnight and were incubated with 0.5 μg/mL of LPS alone for 4 h followed by the addition of 10 μM of

Nigericin for an additional 1 h or 5 μM of staurosporine (Cat#S6942, Sigma) for 4 h to be used as positive controls for inflammasome activation and apoptosis, respectively. Images were acquired using the Keyence BZ-X810 fluorescence microscope (Keyence America, Itasca, IL) at 400X magnification.

Determination of cervical dilation

Nlrp3$^{+/+}$ or *Nlrp3*$^{-/-}$ dams were intra-amniotically injected with 100 ng/25 μL of LPS or 25 μL of PBS in each amniotic sac on 16.5 dpc, and euthanized 16 h post injection. The cervical tissues were collected, photographed, and measured to determine cervical width as a surrogate of cervical dilation, as previously described (7). Briefly, after the isolation of the uterine horns and cervix, we removed the vagina and incised the upper limit of the cervix to isolate the tissue, followed by macroscopic measurement of cervical width using a ruler.

Leukocyte isolation from decidual and uterine tissues

Isolation of leukocytes from decidual or uterine tissues was performed as previously described with brief modifications (13) for immunophenotyping or FACS. Dams were intra-amniotically injected with 100 ng/25 μL of LPS or 25 μL of PBS in each amniotic sac on 16.5 dpc, as described above. Mice were euthanized on 17.5 dpc (16 h after intra-amniotic injection and without anesthesia/ultrasonography), and the decidual and uterine tissues were collected. The decidual and uterine tissues from each dam were pooled prior to digestion. Tissues were gently minced using fine scissors and enzymatically digested with StemPro Accutase Cell Dissociation reagent (Thermo Fisher) for 25 min at 37°C.

Leukocyte suspensions were filtered using a 100-µm cell strainer (Fisherbrand; Fisher Scientific, Fair Lawn, NY) and washed with PBS immediately prior to immunophenotyping or FACS.

Immunophenotyping of leukocytes from decidual and uterine tissues

Leukocytes isolated from the decidua and uterus (one set of pooled tissues from each dam) were incubated with the CD16/CD32 mAb (FcγIII/II receptor; BD Biosciences) for 10 min, followed by the incubation with fluorochrome-conjugated anti-mouse mAbs (Supplemental Table 19, Antibodies for extracellular staining) for 30 min at 4°C in the dark. After washing, the cells were fixed and permeabilized with the BD Cytofix/Cytoperm kit (BD Biosciences) and subsequently incubated with anti-mouse mAbs (Supplemental Table 19, Antibodies for intracellular staining) for 30 min at 4°C in the dark. Following staining, cells were acquired using the BD LSRFortessa flow cytometer (BD Biosciences) and FACSDiva 8.0 software (BD Biosciences). The leukocyte numbers were adjusted according to the number of tissues collected per dam. Heatmaps were generated using log_2-transformed cell counts.

Fluorescence-activated cell sorting and RNA isolation of viable neutrophils and macrophages from decidua and uterus

Leukocytes were isolated from the decidua and uterus as mentioned above. After stained with viability dye, the cells were resuspended in 50 µL of stain buffer (BD Biosciences) and incubated with fluorochrome-conjugated anti-mouse mAbs (Supplemental Table 19, antibodies for cell sorting) for 30 min at 4°C in the dark. The

cells were then washed with stain buffer to remove excess mAbs and resuspended in 500 μL of stain buffer for sorting. Viable CD45+CD11b+Ly6G+F4/80- neutrophils and viable CD45+CD11b+F4/80+Ly6G- macrophages were sorted using the BD FACSMelody cell sorter (BD Biosciences) and BD FACSChorus v1.3 software (BD Biosciences). Sorted cells were collected by centrifugation at 3,000 x g for 10 min at room temperature and resuspended with the RNA Extraction buffer included in the Pico Pure RNA isolation kit (Applied Biosystems by Thermo Fisher Scientific). The cell suspensions were incubated at 42°C for 30 min and centrifuged at 3,000 x g for 2 min at room temperature. The supernatants were stored at −80°C until RNA isolation. Total RNA was isolated using the Pico Pure RNA isolation kit, according to the manufacturer's instructions. RNA concentrations, purity, and integrity were evaluated using the NanoDrop 1000 spectrophotometer and the Bioanalyzer 2100. To obtain sufficient quantities of RNA for RNA-seq, two to four neutrophil samples (biological replicates) from each group were combined. Low-input RNA-seq libraries were constructed by Beijing Genomics Institute (BGI; Wuhan, China) using the Nextera DNA Library Preparation Kit (Illumina, San Diego, CA, USA). The libraries were sequenced on the DNB sequencer (DNBSEQ-G400, BGI) with paired-end 100 base reads. Raw data were provided by BGI.

RNA-seq data analysis

Transcript abundance from RNA-seq reads was quantified with Salmon (14) and used to test for differential expression with a negative binomial distribution model implemented in the DESeq2 (15) package from Bioconductor (16). Genes with a minimum fold change of 1.25-fold and an adjusted *p*-value (*q*-value) of <0.1 were considered

differentially expressed. Venn diagrams were drawn for overlapping sets of up and downregulated genes. The differentially expressed genes (DEGs) for each between-group comparison were used as input for the iPathwayGuide software (ADVAITA Bioinformatics, Ann Arbor, MI, USA) (17-19) to determine the enriched biological processes. Volcano plots were used to display the evidence of differential gene expression between *Nlrp3*$^{+/+}$ dams injected with LPS and *Nlrp3*$^{-/-}$ dams injected with LPS. The top ten enriched Biological Processes (BP) are shown in horizontal bar charts from the most to least significant adjusted *p*-value.

Heterozygous pregnancy models

Nlrp3$^{+/+}$ females were mated with *Nlrp3*$^{-/-}$ males of proven fertility to obtain *Nlrp3*$^{+/+}$ dams carrying *Nlrp3*$^{+/-}$ fetuses, and *Nlrp3*$^{-/-}$ females were mated with *Nlrp3*$^{+/+}$ males of proven fertility to obtain *Nlrp3*$^{-/-}$ dams carrying *Nlrp3*$^{+/-}$ fetuses. These dams were utilized to evaluate pregnancy outcomes in a model of LPS-induced intra-amniotic inflammation, as described above.

Embryo transfer experiments

Eight- to nine-week-old female *Nlrp3*$^{+/+}$ or *Nlrp3*$^{-/-}$ mice underwent superovulation by intraperitoneal injection (i.p.) with 5 IU/100 µL of pregnant mare serum gonadotropin (PMSG; Cat#HOR-272; Prospec, Rehovot, Israel) and 5 IU/100 µL (i.p.) of human chorionic gonadotropin (hCG; Cat#CG-5; Sigma-Aldrich), both dissolved in sterile PBS. The females were mated with a male of the same genotype overnight. The next morning, females with a vaginal plug were utilized as embryo donors. Day 0.5 embryos were

collected and cultured in global media (Cat#LGGG-025; Cooper Surgical, Trumbull, CT, USA) supplemented with 2% bovine serum albumin (BSA, Sigma-Aldrich). To obtain pseudopregnant embryo recipient mice, female *Nlrp3*$^{+/+}$ or *Nlrp3*$^{-/-}$ mice were mated with vasectomized males of the same genotype overnight and checked the next morning for the appearance of a vaginal plug. The pseudopregnant females were used as embryo recipients on 0.5 dpc. The recipient mice were anesthetized with 100 mg/kg ketamine (Ketathesia, Henry Schein, Melville, NY, USA) and 13.5 mg/kg of xylazine (AnaSed, AKORN, Lake Forest, IL, USA) and placed in a prone position. The fur on the back was removed using an electric clipper and the skin was wiped with iodine. A skin incision (approximately 1 cm) was made along the dorsal midline, the skin over the left ovary was pulled back, and the peritoneum was cut to expose the ovary and oviduct. Seven-to-ten embryos from dams of the opposing genotype (*Nlrp3*$^{+/+}$ donor embryos for *Nlrp3*$^{-/-}$ recipients and *vice versa*) were transferred into the oviduct. The surgical procedure was then repeated to expose the right oviduct, and the same number of embryos was transferred. The skin was closed and the recipient mice were placed on a heating pad until they regained full motor function. Their weights were monitored daily, and pregnancy was confirmed by a weight gain of ≥2 grams by 12.5 dpc. The resulting *Nlrp3*$^{+/+}$ dams carrying *Nlrp3*$^{-/-}$ fetuses and *Nlrp3*$^{-/-}$ dams carrying *Nlrp3*$^{+/+}$ fetuses were utilized to evaluated pregnancy outcomes in a model of LPS-induced intra-amniotic inflammation, as described above.

Statistics

Statistical analyses were performed using GraphPad Prism (v9.0.2; GraphPad, San Diego, CA, USA) and the R package (v.3.5.1; https://www.r-project.org/). The Fisher's exact test was used to compare the rates of preterm birth and neonatal mortality. Kaplan–Meier survival curves were used to plot and compare the gestational length data (Gehan-Breslow-Wilcoxon test). The Mann-Whitney U test was used to compare PBS- and LPS-injected dams of each genotype. RNA-seq analysis was performed as described above. A p-value < 0.05 was considered statistically significant.

REFERENCES

1. Gomez-Lopez N, et al. Intra-amniotic administration of lipopolysaccharide induces spontaneous preterm labor and birth in the absence of a body temperature change. *J Matern Fetal Neonatal Med.* 2018;31(4):439-46.
2. Garcia-Flores V, et al. Inflammation-Induced Adverse Pregnancy and Neonatal Outcomes Can Be Improved by the Immunomodulatory Peptide Exendin-4. *Front Immunol.* 2018;9(1291.
3. Faro J, et al. Intra-amniotic inflammation induces preterm birth by activating the NLRP3 inflammasome†. *Biol Reprod.* 2019;100(5):1290-305.
4. Gomez-Lopez N, et al. In vivo T-cell activation by a monoclonal αCD3ε antibody induces preterm labor and birth. *Am J Reprod Immunol.* 2016;76(5):386-90.
5. St Louis D, et al. Invariant NKT Cell Activation Induces Late Preterm Birth That Is Attenuated by Rosiglitazone. *J Immunol.* 2016;196(3):1044-59.
6. Meyer N, et al. High Frequency Ultrasound for the Analysis of Fetal and Placental Development In Vivo. *J Vis Exp.* 2018141).
7. Arenas-Hernandez M, et al. Effector and Activated T Cells Induce Preterm Labor and Birth That Is Prevented by Treatment with Progesterone. *J Immunol.* 2019;202(9):2585-608.
8. Gomez-Lopez N, et al. Regulatory T Cells Play a Role in a Subset of Idiopathic Preterm Labor/Birth and Adverse Neonatal Outcomes. *Cell Rep.* 2020;32(1):107874.

9. Galaz J, et al. A Protocol for Evaluating Vital Signs and Maternal-Fetal Parameters Using High-Resolution Ultrasound in Pregnant Mice. *STAR Protoc.* 2020;1(3):100134.

10. Gomez-Lopez N, et al. Inhibition of the NLRP3 inflammasome can prevent sterile intra-amniotic inflammation, preterm labor/birth, and adverse neonatal outcomesdagger. *Biol Reprod.* 2019;100(5):1306-18.

11. Motomura K, et al. The alarmin interleukin-1alpha causes preterm birth through the NLRP3 inflammasome. *Mol Hum Reprod.* 2020;26(9):712-26.

12. Schneider CA, et al. NIH Image to ImageJ: 25 years of image analysis. *Nat Methods.* 2012;9(7):671-5.

13. Arenas-Hernandez M, et al. Isolation of Leukocytes from the Murine Tissues at the Maternal-Fetal Interface. *J Vis Exp.* 201599):e52866.

14. Patro R, et al. Salmon provides fast and bias-aware quantification of transcript expression. *Nat Methods.* 2017;14(4):417-9.

15. Love MI, et al. Moderated estimation of fold change and dispersion for RNA-seq data with DESeq2. *Genome Biol.* 2014;15(12):550.

16. Gentleman RC, et al. Bioconductor: open software development for computational biology and bioinformatics. *Genome Biol.* 2004;5(10):R80.

17. Draghici S, et al. A systems biology approach for pathway level analysis. *Genome Res.* 2007;17(10):1537-45.

18. Khatri P, et al. In: Rueda L, Mery D, and Kittler J eds. *Lecture Notes in Computer Science*. Berlin: Springer; 2007.

19. Tarca AL, et al. A novel signaling pathway impact analysis. *Bioinformatics.* 2009;25(1):75-82.

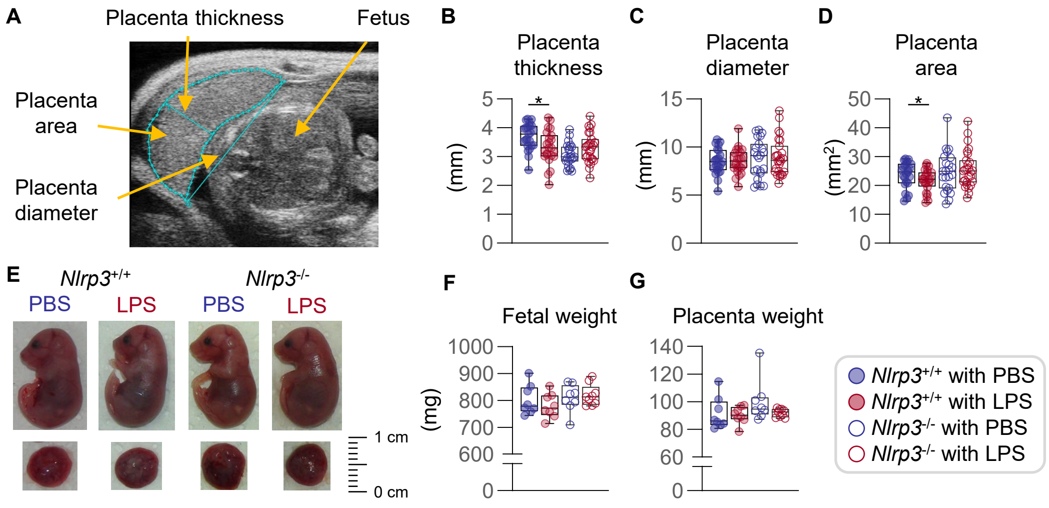

Supplemental Figure 1. Prenatal evaluation of *Nlrp3*$^{-/-}$ mice. Pregnant *Nlrp3*$^{+/+}$ and *Nlrp3*$^{-/-}$ mice were intra-amniotically injected with LPS (100 ng/25 μL) or PBS (n = 8 – 9 per group) in each amniotic sac under ultrasound guidance on 16.5 days *post coitum (*dpc). Ultrasonographic evaluation of the placenta and the measurement of fetal and placental weights were performed on 17.5 dpc. (**A**) Representative ultrasonography image showing placental evaluation. (**B**) Placental thickness. (**C**) Placental diameter. (**D**) Placental area. (**E**) Representative fetal and placental images. (**F**) Fetal weights. (**G**) Placental weights. The *p*-values of the comparisons between PBS- and LPS-injected dams of each genotype were determined by Mann-Whitney U-tests. *, $p < 0.05$.

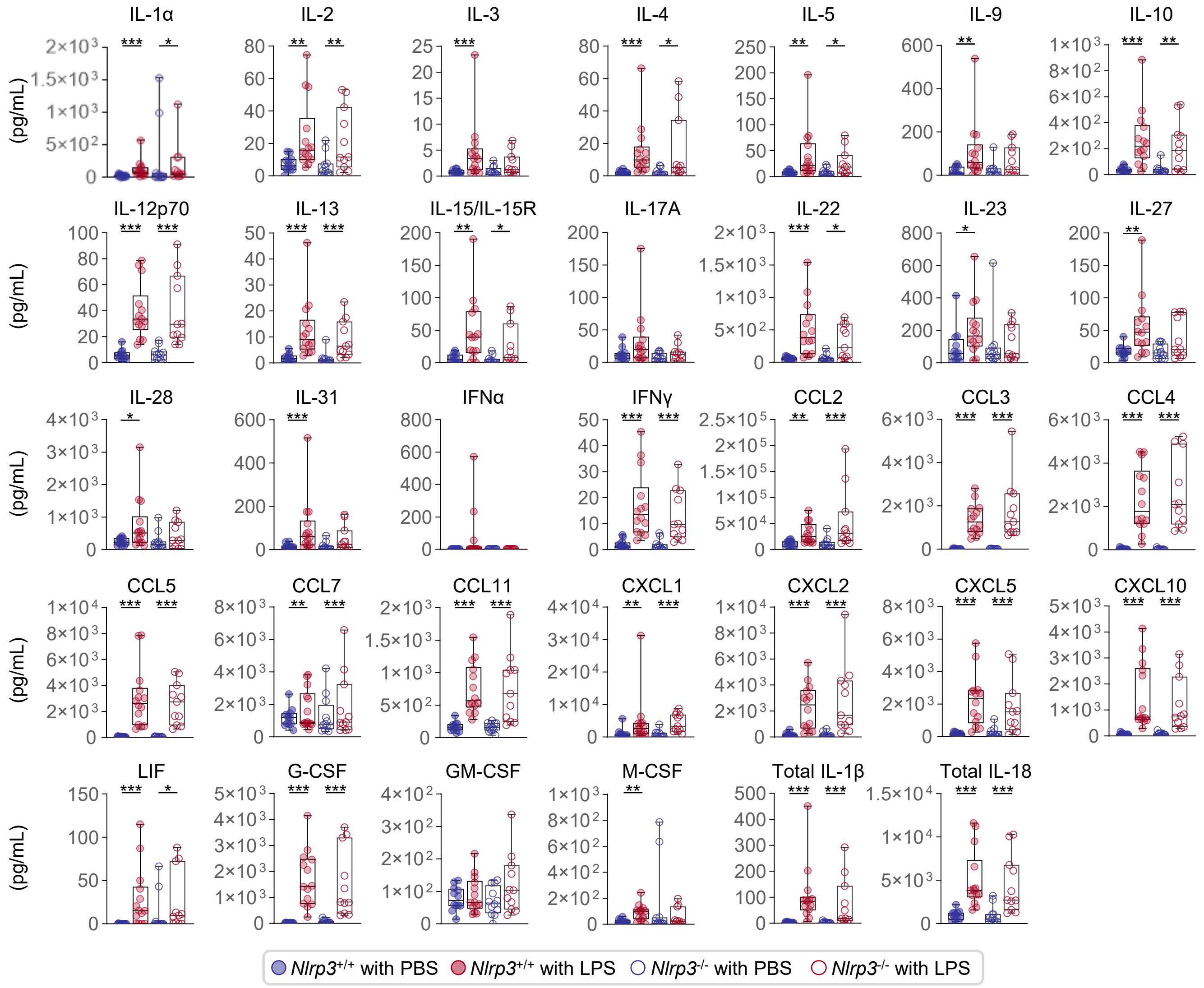

Supplemental Figure 2. Amniotic fluid concentrations of inflammatory mediators. Pregnant *Nlrp3*$^{+/+}$ and *Nlrp3*$^{-/-}$ mice were intra-amniotically injected with LPS (100 ng/25 μL) or PBS in each amniotic sac under ultrasound guidance on 16.5 days *post coitum* (dpc). Amniotic fluid samples were collected on 17.5 dpc (n = 11 - 14 per group). The *p*-values of the comparisons between PBS- and LPS-injected dams of each genotype were determined by Mann-Whitney U-test. *, $p < 0.05$; **, $p < 0.01$; ***, $p < 0.001$.

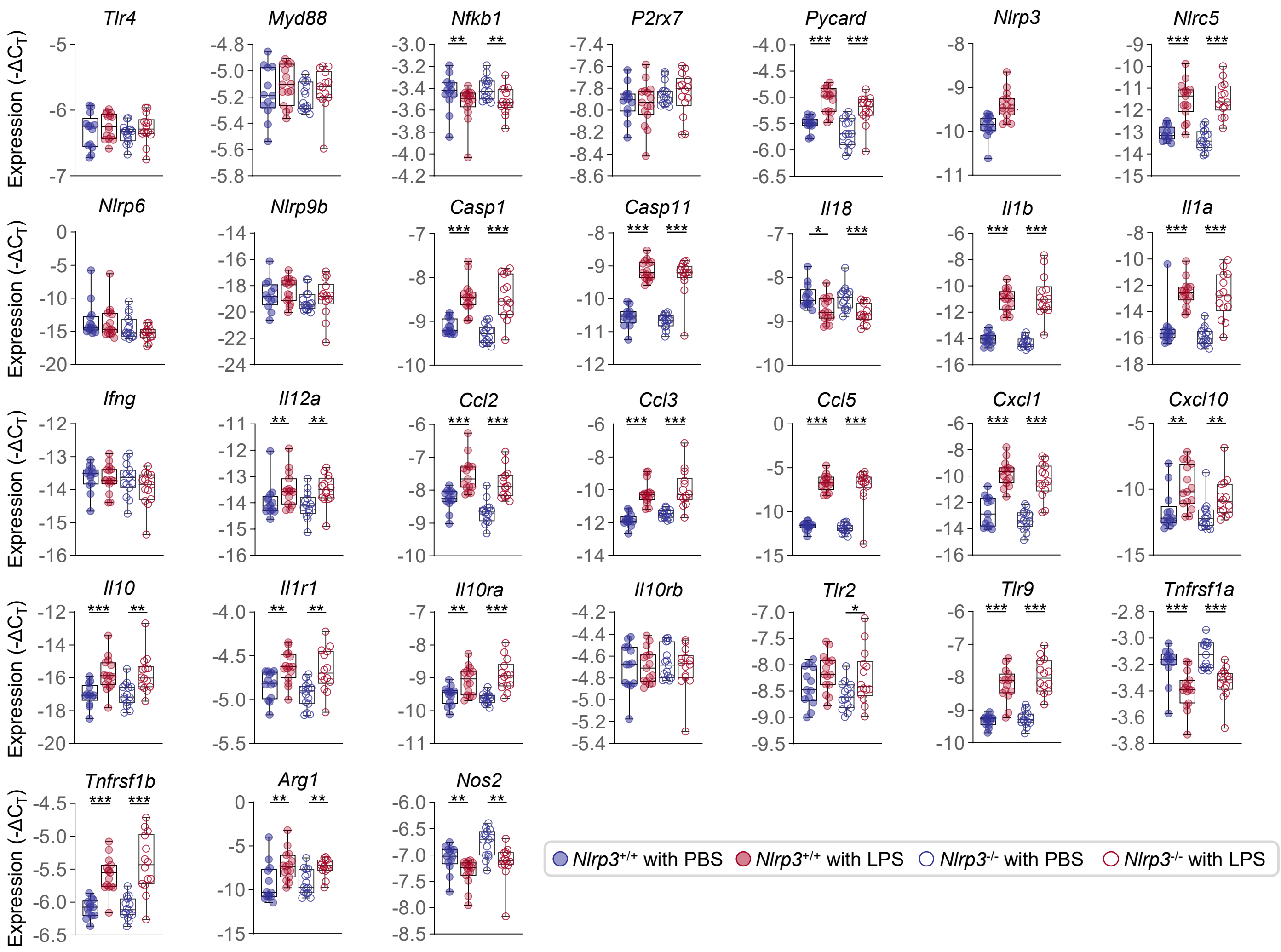

Supplemental Figure 3. Gene expression in the fetal lung. Pregnant *Nlrp3*$^{+/+}$ and *Nlrp3*$^{-/-}$ mice were intra-amniotically injected with LPS (100 ng/25 µL) or PBS in each amniotic sac under ultrasound guidance on 16.5 days *post coitum* (dpc). Fetal lung was collected on 17.5 dpc (n = 13 - 15 per group). The *p*-values of the comparisons between PBS- and LPS-injected dams of each genotype were determined by Mann-Whitney U-test. *, $p < 0.05$; **, $p < 0.01$; ***, $p < 0.001$.

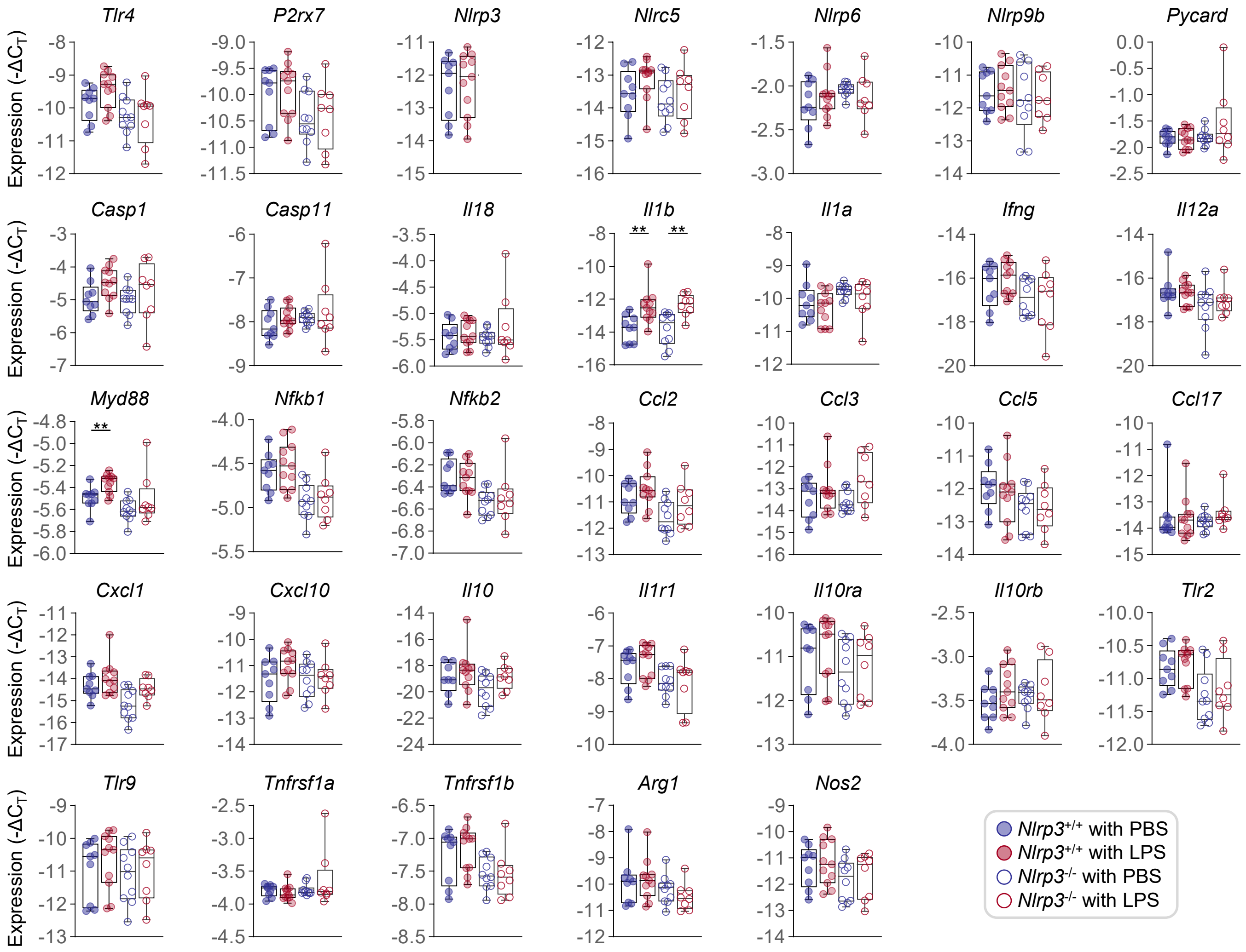

Supplemental Figure 4. Gene expression in the fetal intestine. Pregnant *Nlrp3*$^{+/+}$ and *Nlrp3*$^{-/-}$ mice were intra-amniotically injected with LPS (100 ng/25 µL) or PBS in each amniotic sac under ultrasound guidance on 16.5 days *post coitum* (dpc). Fetal intestine was collected on 17.5 dpc (n = 13 - 15 per group). The *p*-values of the comparisons between PBS- and LPS-injected dams of each genotype were determined by Mann-Whitney U-test. **, $p < 0.01$.

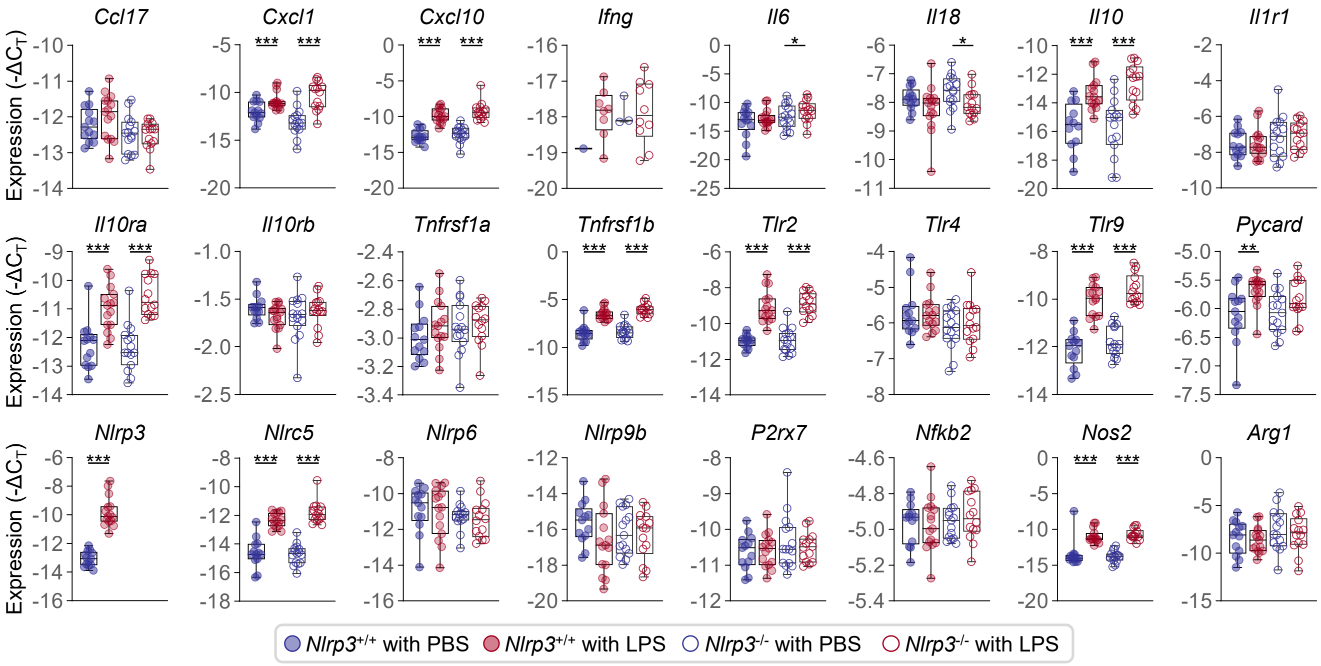

Supplemental Figure 5. Gene expression in the fetal membranes. Pregnant *Nlrp3*$^{+/+}$ and *Nlrp3*$^{-/-}$ mice were intra-amniotically injected with LPS (100 ng/25 μL) or PBS in each amniotic sac under ultrasound guidance on 16.5 days *post coitum* (dpc). Fetal membranes were collected on 17.5 dpc (n = 13 - 15 per group). The *p*-values of the comparisons between PBS- and LPS-injected dams of each genotype were determined by Mann-Whitney U-test. *, $p < 0.05$; **, $p < 0.01$; ***, $p < 0.001$.

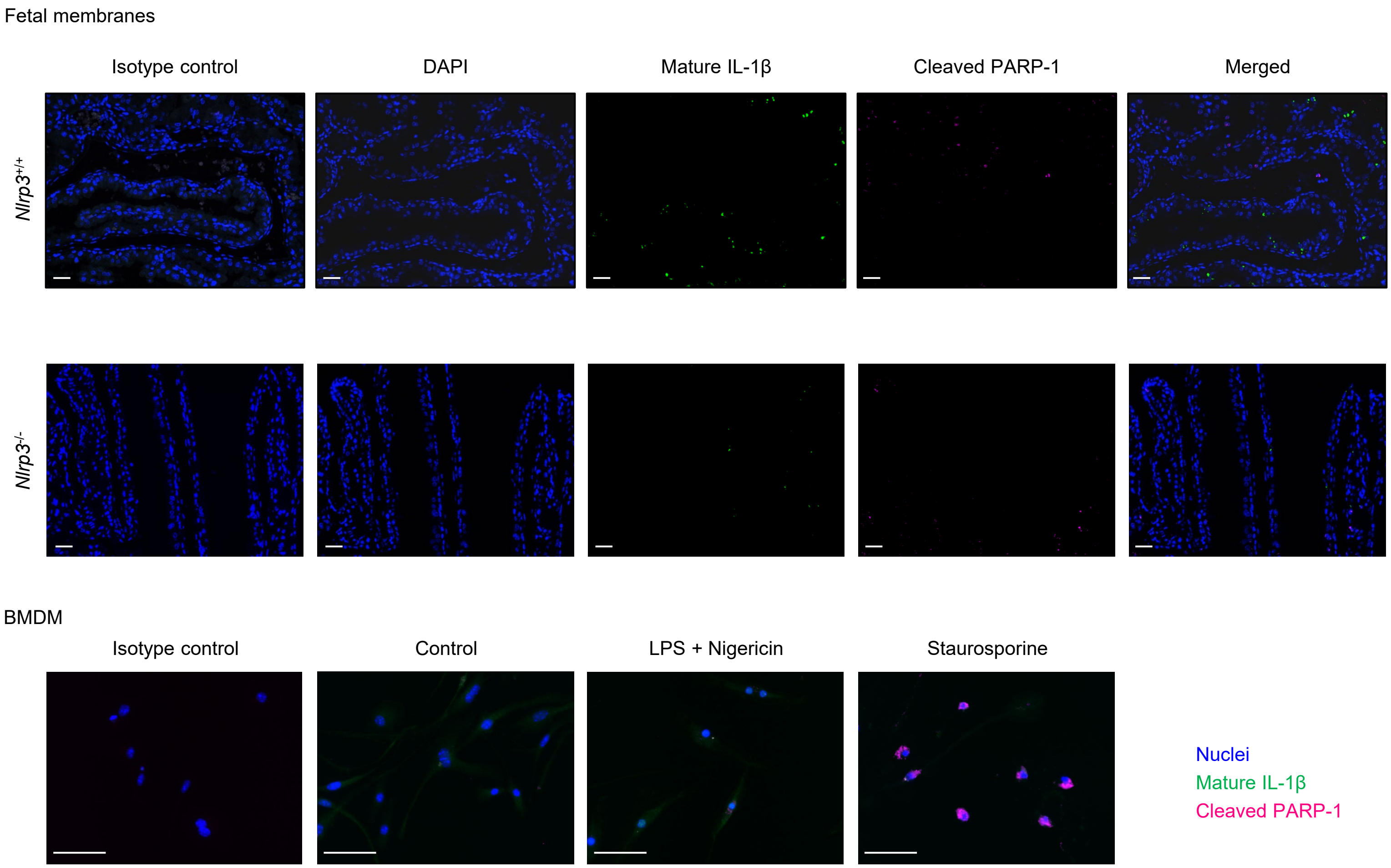

Supplemental Figure 6. Localization of mature IL-1β and cleaved PARP-1 in the fetal membranes. Pregnant *Nlrp3*$^{+/+}$ and *Nlrp3*$^{-/-}$ mice were intra-amniotically injected with LPS (100 ng/25 µL) in each amniotic sac under ultrasound guidance on 16.5 days *post coitum* (dpc). Fetal membranes were collected on 17.5 dpc (n = 2 – 3 per group). Representative immunofluorescence staining of mature IL-1β (green) and cleaved PARP-1 (magenta). Images were taken at 40X magnification. Bone marrow derived macrophages (BMDMs) incubated without treatment (control), with LPS + Nigericin (inflammasome activation), or with staurosporine (apoptosis) are shown as controls. Images were taken at 400X magnification. Scale bars = 50 µm.

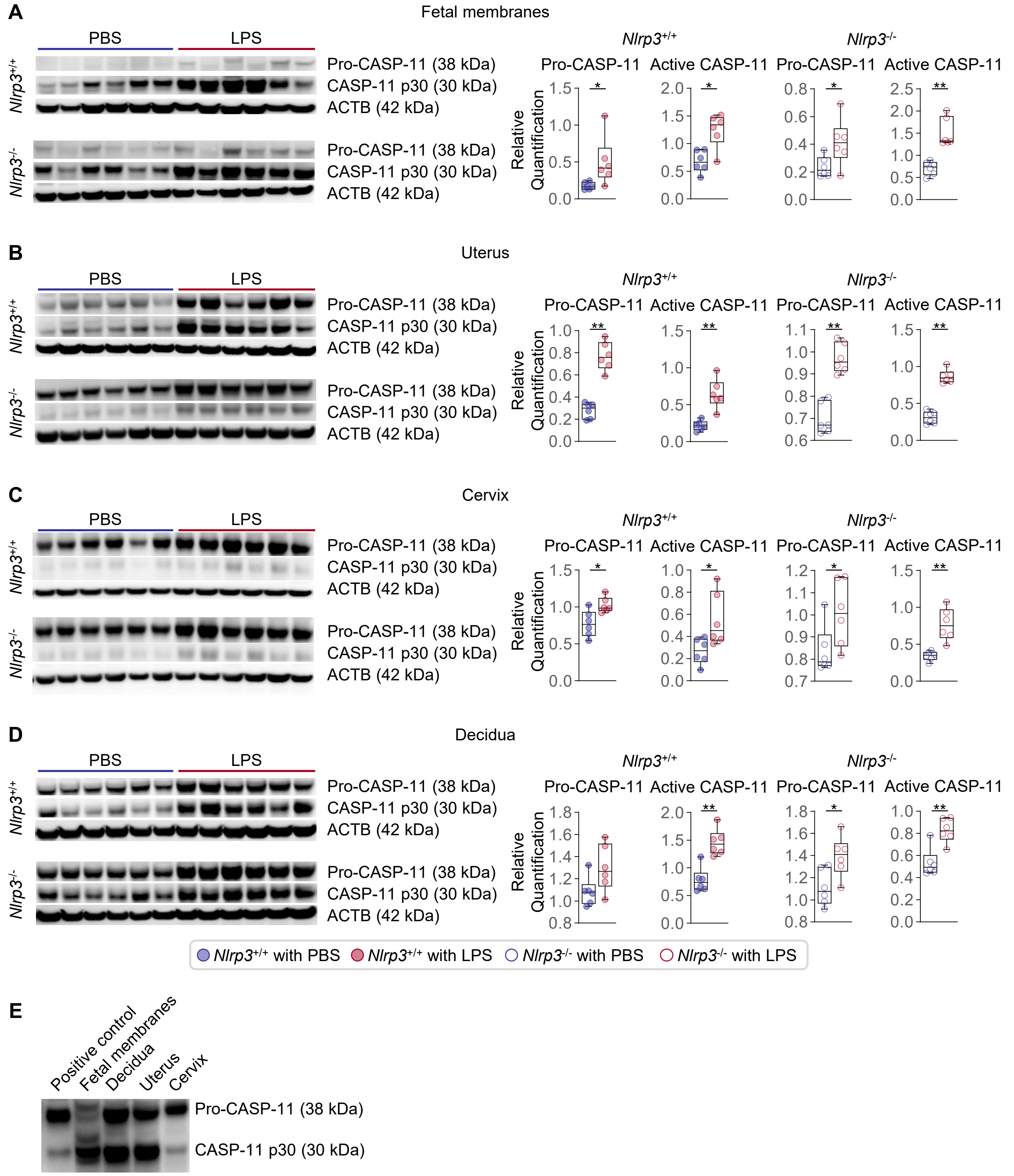

Supplemental Figure 7. Caspase-11 activation in the fetal and maternal tissues. Pregnant *Nlrp3*$^{+/+}$ and *Nlrp3*$^{-/-}$ mice were intra-amniotically injected with LPS (100 ng/25 μL) or PBS in each amniotic sac under ultrasound guidance on 16.5 days *post coitum* (dpc). The fetal membranes, uterus, cervix, and decidua were collected on 17.5 dpc (n = 6 per group). (**A**-**D**) Immunoblotting of caspase (CASP)-11 p38 and p30 in the (**A**) fetal membranes, (**B**) uterus, (**C**) cervix, and (**D**) decidua. The expression was normalized by ACTB and shown as relative quantification. (**E**) CASP-11 immunoblotting of the positive control and representative tissue samples (fetal membranes, decidua, uterus, and cervix) from *Nlrp3*$^{+/+}$ mice intra-amniotically injected with LPS. Data are shown as box and whisker plots where midlines indicate medians, boxes indicate interquartile ranges, and whiskers indicate minimum and maximum values. The *p*-values of the comparisons between PBS- and LPS-injected dams of each genotype were determined by Mann-Whitney U-test. *, $p < 0.05$; **, $p < 0.01$.

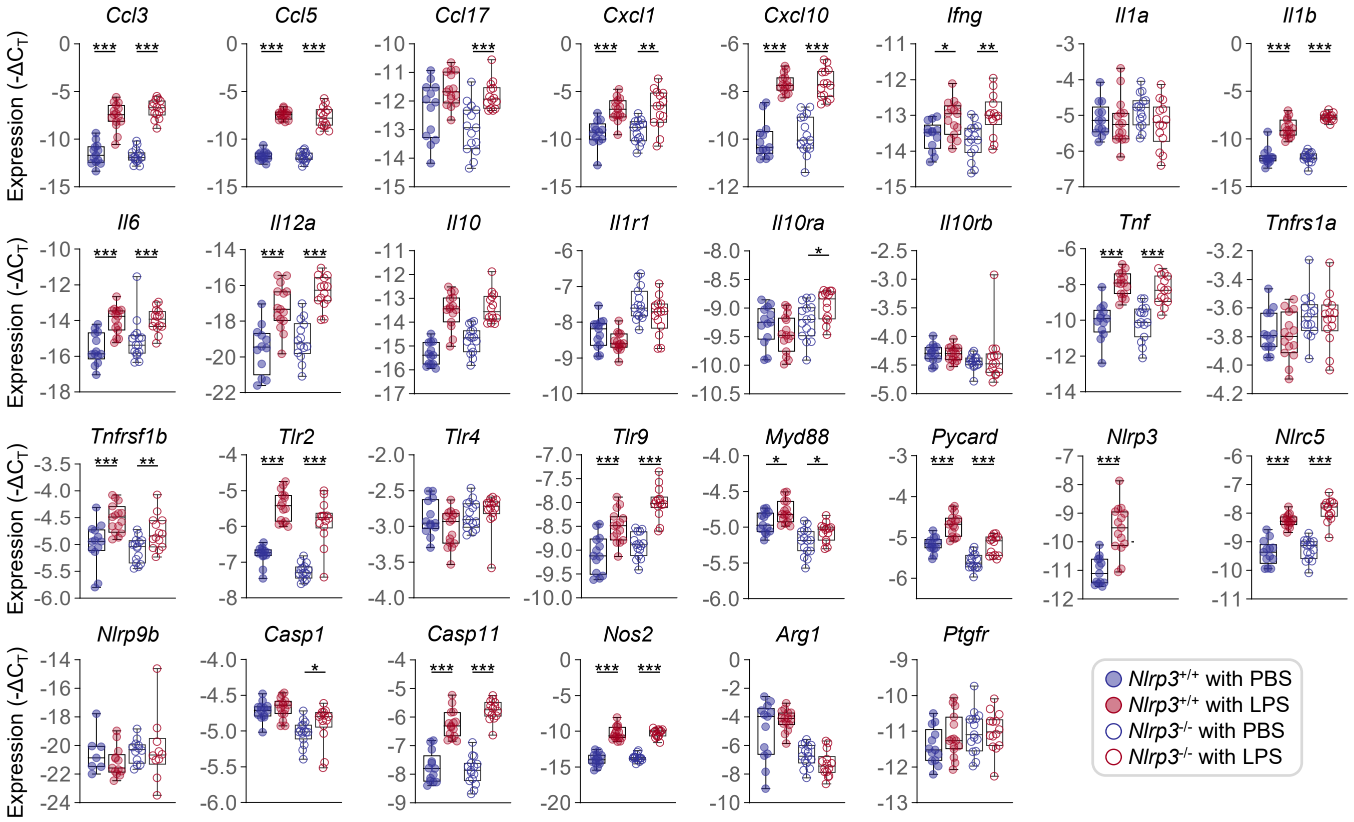

Supplemental Figure 8. Gene expression in the uterus. Pregnant *Nlrp3*$^{+/+}$ and *Nlrp3*$^{-/-}$ mice were intra-amniotically injected with LPS (100 ng/25 µL) or PBS in each amniotic sac under ultrasound guidance on 16.5 days *post coitum* (dpc). Uterine tissues were collected on 17.5 dpc (n = 13-15 per group). The *p*-values of the comparisons between PBS- and LPS-injected dams of each genotype were determined by Mann-Whitney U-test. *, $p < 0.05$; **, $p < 0.01$; ***, $p < 0.001$.

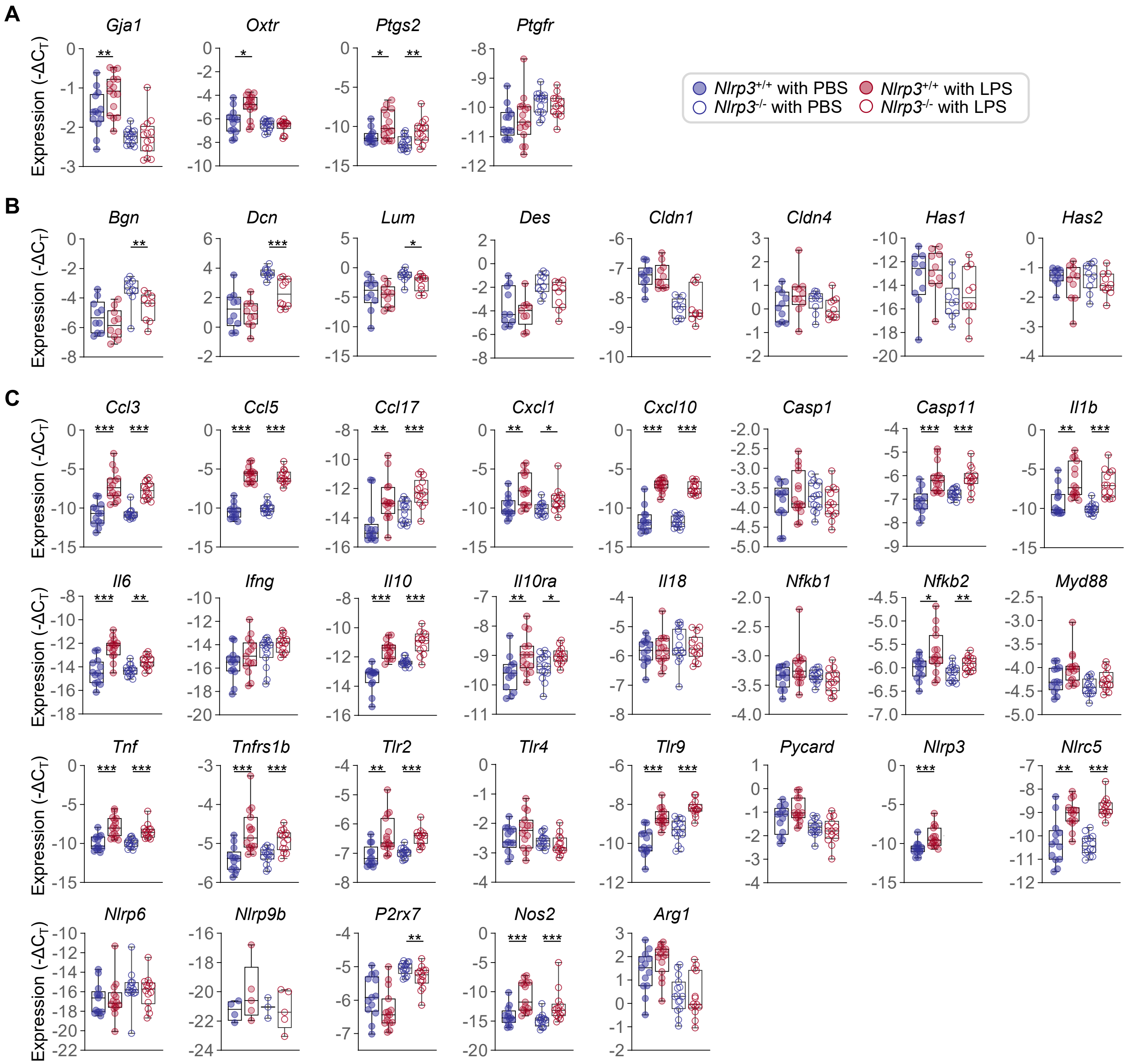

Supplemental Figure 9. Gene expression in the cervix. Pregnant *Nlrp3*$^{+/+}$ and *Nlrp3*$^{-/-}$ mice were intra-amniotically injected with LPS (100 ng/25 μL) or PBS in each amniotic sac under ultrasound guidance on 16.5 days *post coitum* (dpc). Cervical tissues were collected on 17.5 dpc (n = 10 - 15 per group). Expression of (**A**) contractility-associated, (**B**) cervical remodeling-associated, and (**C**) inflammation-associated genes. The *p*-values of the comparisons between PBS- and LPS-injected dams of each genotype were determined by Mann-Whitney U-test. *, $p < 0.05$; **, $p < 0.01$; ***, $p < 0.001$.

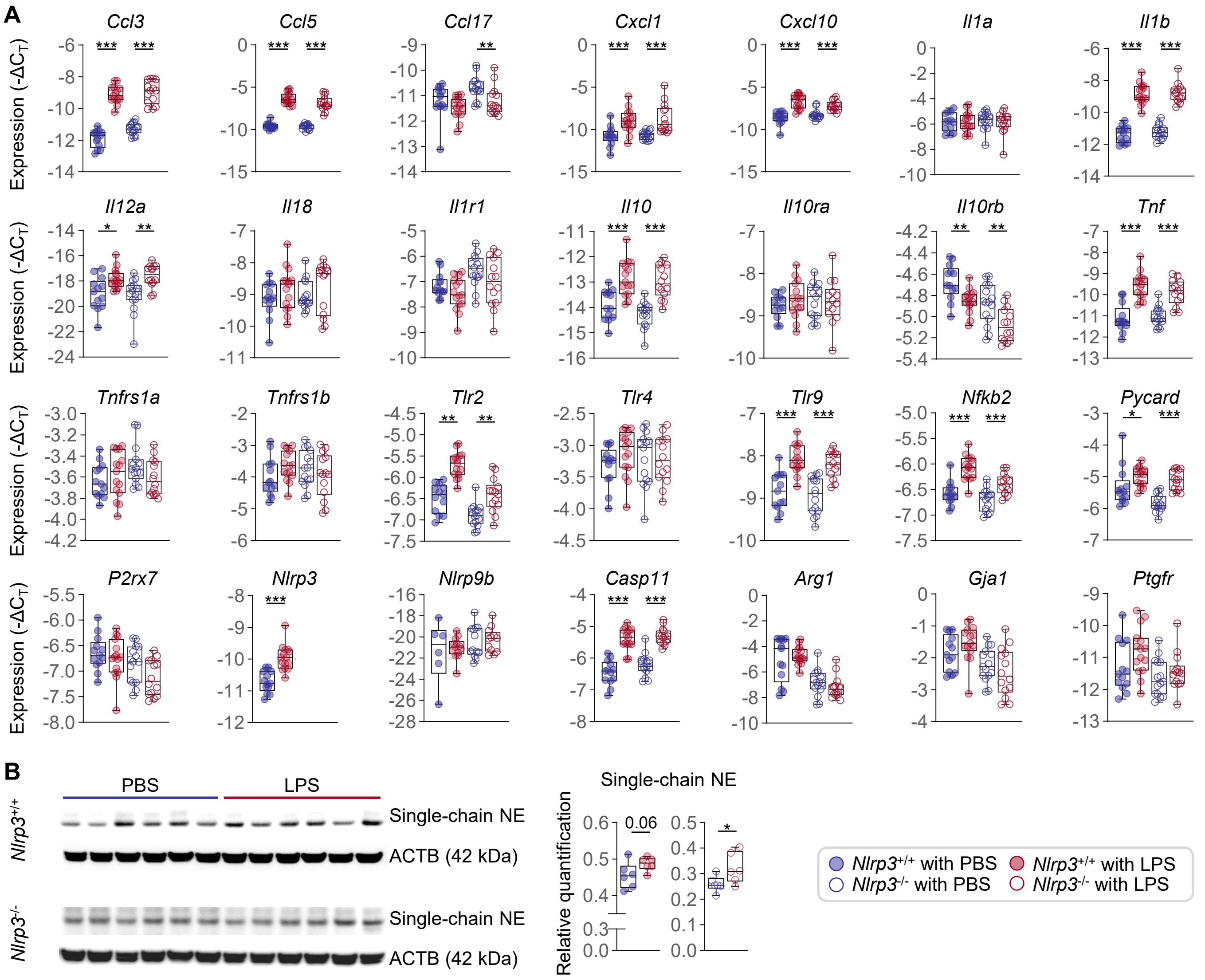

Supplemental Figure 10. Inflammatory mediator expression in the decidua. Pregnant *Nlrp3*$^{+/+}$ and *Nlrp3*$^{-/-}$ mice were intra-amniotically injected with LPS (100 ng/25 μL) or PBS in each amniotic sac under ultrasound guidance on 16.5 days *post coitum* (dpc). Decidua basalis tissues were collected on 17.5 dpc. (**A**) Expression of inflammation-associated genes in the decidua (n = 13 - 15 per group). The *p*-values of the comparisons between PBS- and LPS-injected dams of each genotype were determined by Mann-Whitney U-test. (**B**) Immunoblotting of single-chain neutrophil elastase (NE) in the decidua (n = 6 per group). The expression was normalized by ACTB and shown as relative quantification. The *p*-values of the comparisons between PBS- and LPS-injected dams of each genotype were determined by Mann-Whitney U-test. *, $p < 0.05$; **, $p < 0.01$; ***, $p < 0.001$.

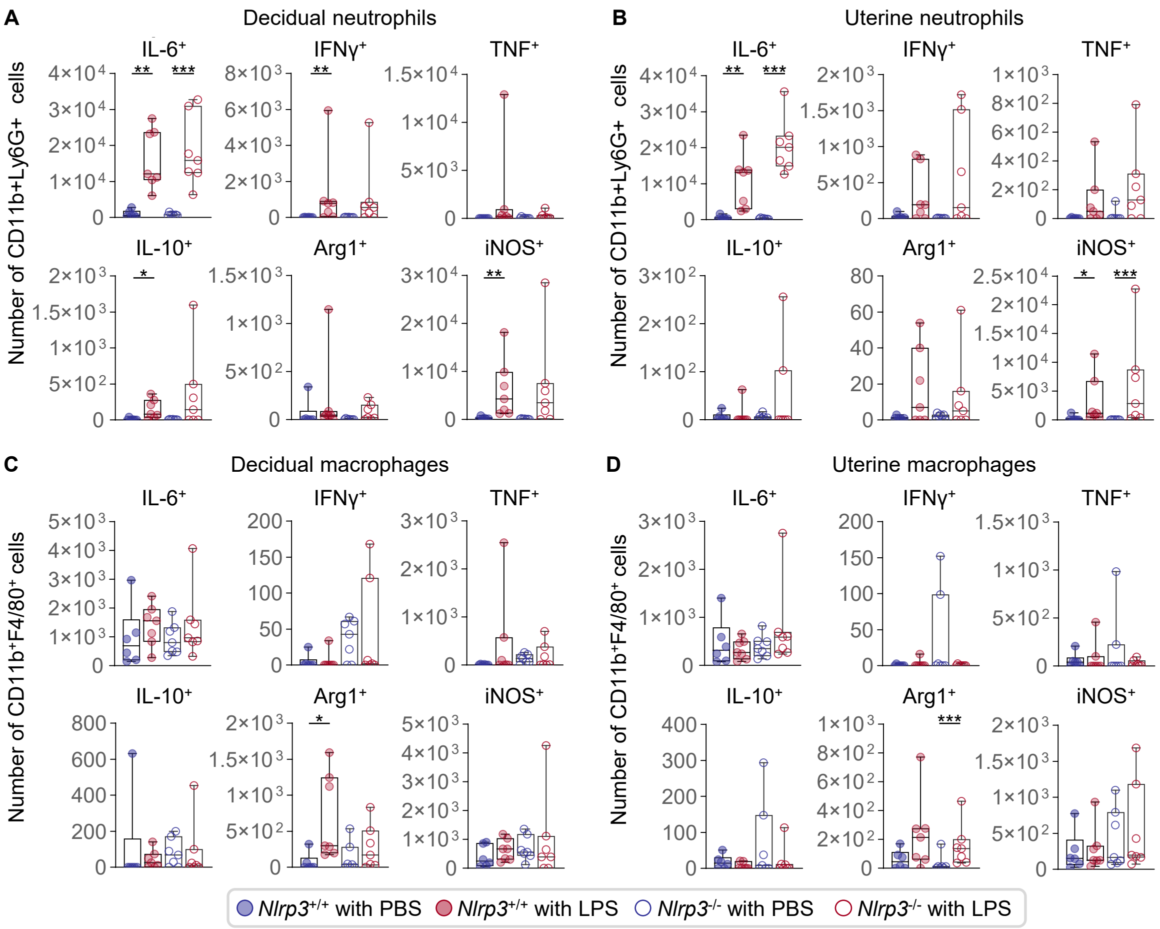

Supplemental Figure 11. Inflammatory mediator expression by decidual and uterine neutrophils and macrophages. Pregnant *Nlrp3*$^{+/+}$ and *Nlrp3*$^{-/-}$ mice were intra-amniotically injected with LPS (100 ng/25 μL) or PBS in each amniotic sac under ultrasound guidance on 16.5 days *post coitum* (dpc). Decidual and uterine tissues were collected on 17.5 dpc (n = 6 - 7 per group). (**A**) Absolute numbers of decidual neutrophils expressing IL-6, IFNγ, TNF, IL-10, Arginase-1 (Arg1), or iNOS. (**B**) Absolute numbers of uterine neutrophils expressing IL-6, IFNγ, TNF, IL-10, Arg1, or iNOS. (**C**) Absolute numbers of decidual macrophages expressing IL-6, IFNγ, TNF, IL-10, Arg1, or iNOS. (**D**) Absolute numbers of uterine macrophages expressing IL-6, IFNγ, TNF, IL-10, Arg1, or iNOS. The *p*-values of the comparisons between PBS- and LPS-injected dams of each genotype were determined by Mann-Whitney U-test. *, $p < 0.05$; **, $p < 0.01$; ***, $p < 0.001$.

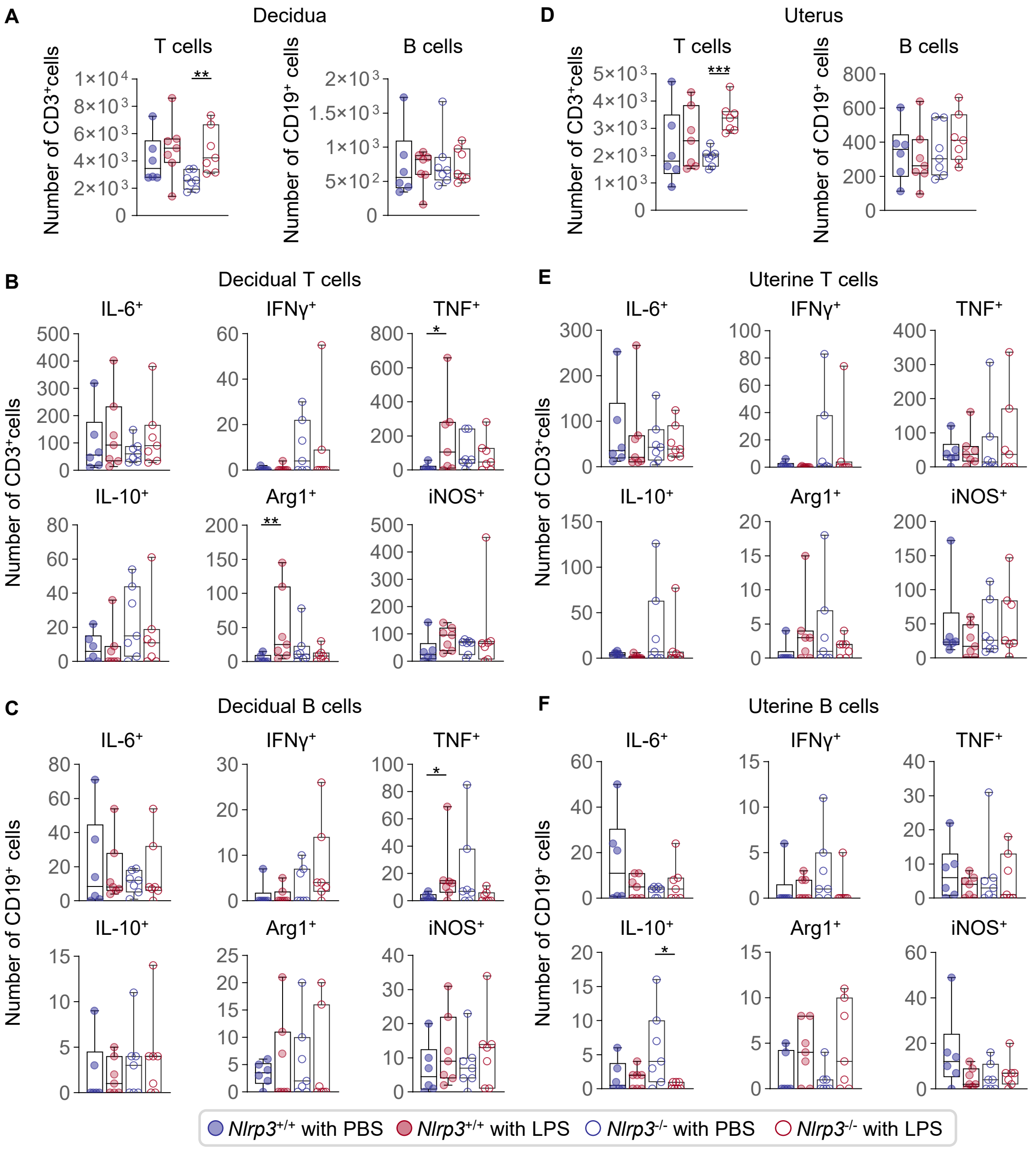

Supplemental Figure 12. Inflammatory mediator expression by decidual and uterine T cells and B cells. Pregnant *Nlrp3*$^{+/+}$ and *Nlrp3*$^{-/-}$ mice were intra-amniotically injected with LPS (100 ng/25 μL) or PBS in each amniotic sac under ultrasound guidance on 16.5 days *post coitum* (dpc). Decidual and uterine tissues were collected on 17.5 dpc (n = 6 - 7 per group). (**A**) Absolute numbers of decidual T cells and B cells. (**B**) Absolute numbers of decidual T cells expressing IL-6, IFNγ, TNF, IL-10, Arginase-1 (Arg1), and iNOS. (**C**) Absolute numbers of decidual B cells expressing IL-6, IFNγ, TNF, IL-10, Arg1, and iNOS. (**D**) Absolute numbers of uterine T cells and B cells. (**E**) Absolute numbers of uterine T cells expressing IL-6, IFNγ, TNF, IL-10, Arg1, and iNOS. (**F**) Absolute numbers of uterine B cells expressing IL-6, IFNγ, TNF, IL-10, Arg1, and iNOS. The *p*-values of the comparisons between PBS- and LPS-injected dams of each genotype were determined by Mann-Whitney U-test. *, $p < 0.05$; **, $p < 0.01$; ***, $p < 0.001$.

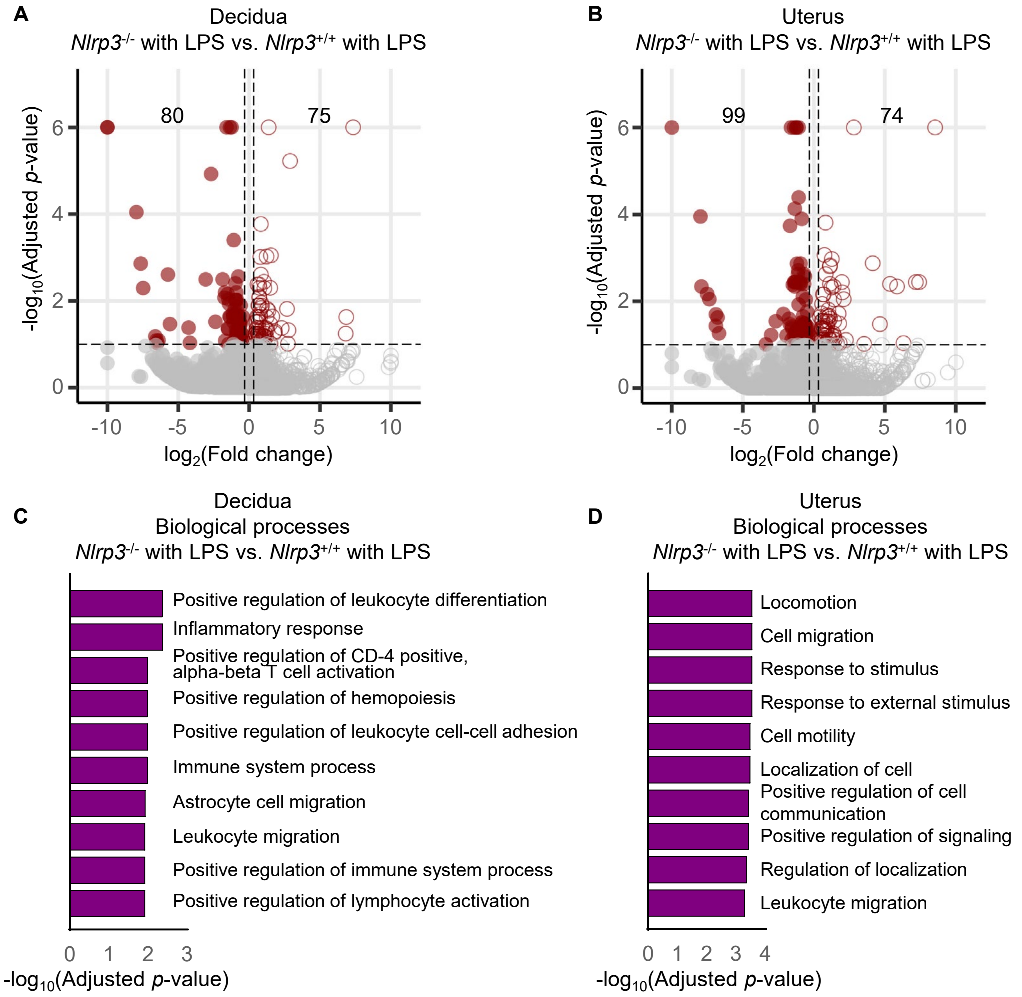

Supplemental Figure 13. Transcriptomic changes in decidual and uterine macrophages. Pregnant *Nlrp3*$^{+/+}$ and *Nlrp3*$^{-/-}$ mice were intra-amniotically injected with LPS (100 ng/25 µL) or PBS in each amniotic sac under ultrasound guidance on 16.5 days *post coitum* (dpc) (n = 3 each). Decidual and uterine tissues were collected on 17.5 dpc. Macrophages were sorted from these tissues by FACS. Macrophage transcriptomes were obtained by RNA-seq. (**A** and **B**) Volcano plots showing the DEGs in (**A**) decidual and (**B**) uterine macrophages from *Nlrp3*$^{+/+}$ and *Nlrp3*$^{-/-}$ dams injected with LPS. (**C** and **D**) Biological processes (BP) enriched in the comparison between (**C**) decidual and (**D**) uterine macrophages from *Nlrp3*$^{+/+}$ and *Nlrp3*$^{-/-}$ dams injected with LPS. Hypergeometric distribution was used to test for significance in the case of BP. The *p*-values for BP were adjusted by false discovery rate.

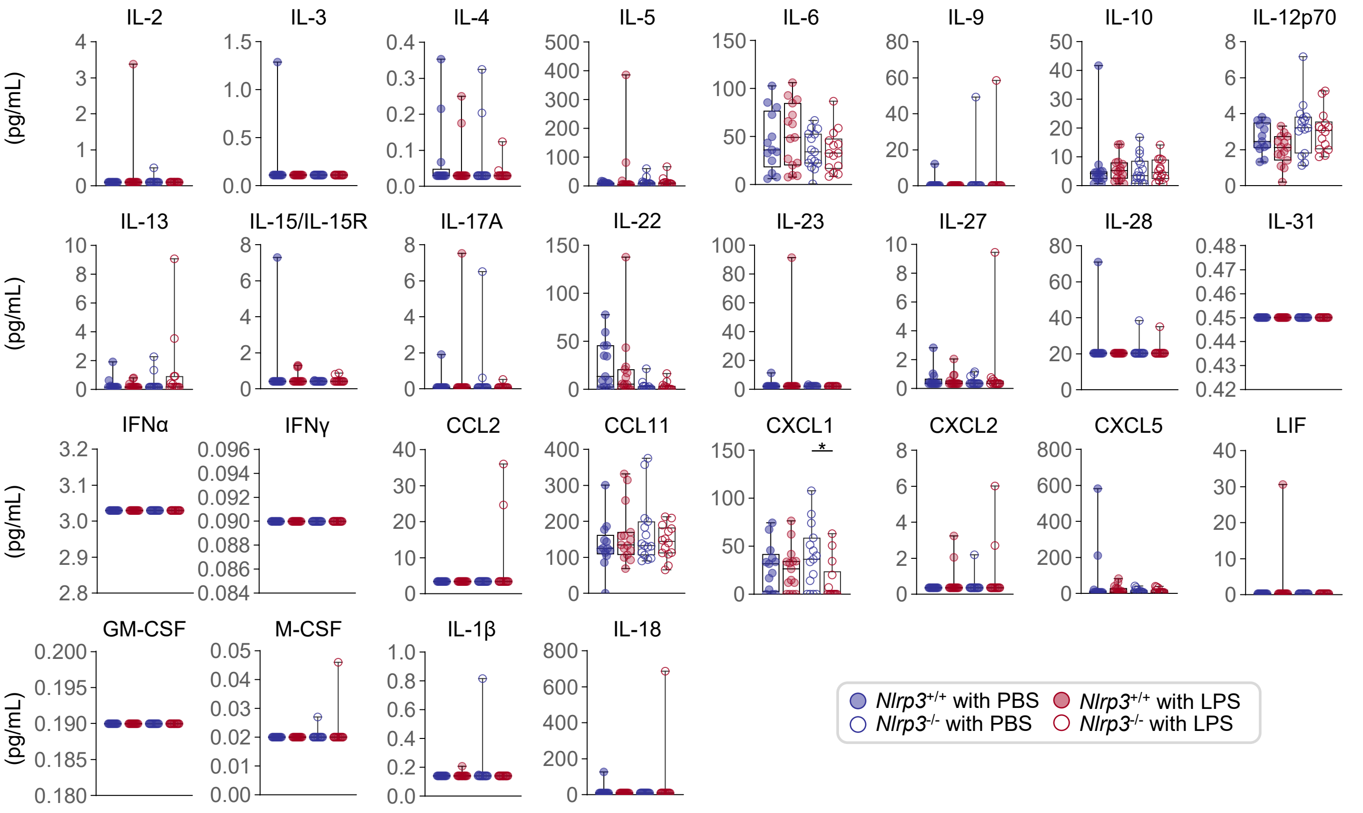

Supplemental Figure 14. Maternal plasma concentrations of inflammatory mediators. Pregnant *Nlrp3*$^{+/+}$ and *Nlrp3*$^{-/-}$ mice were intra-amniotically injected with LPS (100 ng/25 µL) or PBS in each amniotic sac under ultrasound guidance on 16.5 days *post coitum* (dpc). Maternal plasma was collected on 17.5 dpc (n = 13 - 15 per group). The *p*-values of the comparisons between PBS- and LPS-injected dams of each genotype were determined by Mann-Whitney U-test. *, $p < 0.05$.

Supplemental Table 17. Sensitivities for the Luminex assay-based measurements of immune mediators

Immune mediator	Sensitivity (pg/mL)
IFNα	3.03
IFNγ	0.09
IL-12p70	0.21
IL-1β	0.14
IL-2	0.10
TNF	0.39
GM-CSF	0.19
IL-18	9.95
IL-17A	0.08
IL-22	0.24
IL-23	2.21
IL-27	0.34
IL-9	0.28
IL-15/IL-15R	0.42
IL-13	0.16
IL-4	0.03
IL-5	0.32
IL-6	0.21
IL-10	0.69
Eotaxin/CCL11	0.01
IL-28	20.31
IL-3	0.11
LIF	0.28
IL-1α	0.32
IL-31	0.45
GRO-α/CXCL1	0.05
MIP-1α/CCL3	0.13
IP-10/CXCL10	0.26
MCP-1/CCL2	3.43
MCP-3/CCL7	0.15
MIP-1β/CCL4	1.16
MIP-2/CXCL2	0.37
RANTES/CCL5	0.35
G-CSF	0.19
M-CSF	0.02
ENA-78/CXCL5	5.67

Supplemental Table 18. Taqman assays utilized for RT-qPCR

**Name**	**Symbol**	**Assay ID**
Actin, beta	*Actb*	Mm04394036_g1
Glucuronidase, beta	*Gusb*	Mm01197698_m1
Glyceraldehyde-3-phosphate dehydrogenase	*Gapdh*	Mm99999915_g1
Heat shock protein 90 alpha (cytosolic), class B member 1	*Hsp90ab1*	Mm00833431_g1
Arginase, liver	*Arg1*	Mm00475988_m1
Biglycan	*Bgn*	Mm01191753_m1
Caspase 1	*Casp1*	Mm00438023_m1
Caspase 11 (caspase 4)	*Casp11*	Mm00432304_m1
Chemokine (C-C motif) ligand 2	*Ccl2*	Mm00441242_m1
Chemokine (C-C motif) ligand 3	*Ccl3*	Mm00441259_g1
Chemokine (C-C motif) ligand 5	*Ccl5*	Mm01302427_m1
Chemokine (C-C motif) ligand 17	*Ccl17*	Mm01244826_g1
Chemokine (C-X-C motif) ligand 1	*Cxcl1*	Mm04207460_m1
Chemokine (C-X-C motif) ligand 10	*Cxcl10*	Mm00445235_m1
Claudin 1	*Cldn1*	Mm01342184_m1
Claudin 4	*Cldn4*	Mm00515514_s1
Decorin	*Dcn*	Mm00514535_m1
Desmin	*Des*	Mm00802455_m1
Hyaluronate Synthase 1	*Has1*	Mm03048195_m1
Hyaluronate Synthase 2	*Has2*	Mm00515089_m1
Interferon gamma	*Ifng*	Mm01168134_m1
Interleukin-10	*Il10*	Mm01288386_m1
Interleukin-10 receptor alpha	*Il10ra*	Mm00434151_m1
Interleukin-10 receptor beta	*Il10rb*	Mm00434157_m1
Interleukin-12a	*Il12a*	Mm00434169_m1
Interleukin-18	*Il18*	Mm00434226_m1
Interleukin-1 alpha	*Il1a*	Mm00439620_m1
Interleukin-1 beta	*Il1b*	Mm00434228_m1
Interleukin-1 receptor type 1	*Il1r1*	Mm00434237_m1
Interleukin-6	*Il6*	Mm00446190_m1
Lumican	*Lum*	Mm01248292_m1
Myeloid differentiation primary response gene 88	*Myd88*	Mm00440338_m1
Nuclear factor of kappa light polypeptide gene enhancer in B cells 1	*Nfkb1*	Mm00476361_m1
Nuclear factor of kappa light polypeptide gene enhancer in B cells 2	*Nfkb2*	Mm00479807_m1
NLR family, CARD domain containing 5	*Nlrc5*	Mm01243039_m1
NLR family, pyrin domain containing 3	*Nlrp3*	Mm00840904_m1
NLR family, pyrin domain containing 6	*Nlrp6*	Mm00460229_m1
NLR family, pyrin domain containing 9B	*Nlrp9b*	Mm01312681_g1
Nitric oxide synthase 2, inducible	*Nos2*	Mm00440502_m1
Purinergic receptor P2X, ligand-gated ion channel, 7	*P2rx7*	Mm01199500_m1

PYD and CARD domain containing	*Pycard*	Mm00445747_g1
Toll-like receptor 2	*Tlr2*	Mm00442346_m1
Toll-like receptor 4	*Tlr4*	Mm00445273_m1
Toll-like receptor 9	*Tlr9*	Mm00446193_m1
Tumor necrosis factor	*Tnf*	Mm00443258_m1
Tumor necrosis factor receptor superfamily member 1a	*Tnfrsf1a1*	Mm00441883_g1
Tumor necrosis factor receptor superfamily member 1b	*Tnfrsf1b*	Mm00441889_m1
Oxytocin receptor	*Oxtr*	Mm01182684_m1
Gap junction protein, alpha 1	*Gja1*	Mm00439105_m1
Prostaglandin F receptor	*Ptgfr*	Mm00436055_m1
Prostaglandin-endoperoxide synthase 2	*Ptgs2*	Mm00478374_m1

Supplemental Table 19. Antibodies utilized for immunophenotyping and fluorescence-activated cell sorting

Antigen/isotype	Fluorochrome	Clone	Company	Catalog number
Immunophenotyping				
Antibodies for extracellular staining				
CD45	AF700	30-F11	BD Biosciences	560510
CD11b	BV737	M1/70	BD Biosciences	612800
F4/80	APC-eFluor780	BM8	eBioscience	47-4801-82
Ly6G	BV395	1A8	BD Biosciences	563978
CD3	PE-CF594	145-2C11	BD Biosciences	562286
CD19	BV421	1D3	BD Biosciences	562701
Viability	BV510		BD Biosciences	564406
Antibodies for intracellular staining				
iNOS	PE	CXNFT	eBioscience	12-5920-82
Arg1	FITC	Polyclonal	R&D	IC5868F
IFNγ	BV786	XMG1.2	BD Biosciences	563773
IL-10	BV605	JES5-16E3	BD Biosciences	564082
TNF	PECy7	MP6-XT22	BD Biosciences	561041
IL-6	APC	MP5-20F3	BD Biosciences	561367
Rat IgG2a, κ	PE	eBR2a	eBioscience	12-4321-82
Rat IgG2b	FITC	141945	R&D	IC013F
Rat IgG1, κ	BV786	R3-34	BD Biosciences	563847
Rat IgG2b, κ	BV605	R35-38	BD Biosciences	563145
Rat IgG1, κ	PECy7	R3-34	BD Biosciences	557645
Rat IgG1, κ	APC	R3-34	BD Biosciences	554686
Antibodies for cell sorting				
CD45	BV421	30-F11	BD Biosciences	563890
CD11b	APC Cy7	M1/70	BD Biosciences	557657
Ly6G	APC	1A8	BD Biosciences	560599
F4/80	PE	BM8	eBioscience	12-4801-82
Viability	BV510		BD Biosciences	564406
